# Post-Embryonic Nerve-Associated Precursors to Adult Pigment Cells: Genetic Requirements and Dynamics of Morphogenesis and Differentiation

**DOI:** 10.1371/journal.pgen.1002044

**Published:** 2011-05-19

**Authors:** Erine H. Budi, Larissa B. Patterson, David M. Parichy

**Affiliations:** 1 Department of Biology, University of Washington, Seattle, Washington, United States of America; 2 Graduate Program in Molecular and Cellular Biology, University of Washington, Seattle, Washington, United States of America; 3 Graduate Program in Biology, University of Washington, Seattle, Washington, United States of America; Stanford University School of Medicine, United States of America

## Abstract

The pigment cells of vertebrates serve a variety of functions and generate a stunning variety of patterns. These cells are also implicated in human pathologies including melanoma. Whereas the events of pigment cell development have been studied extensively in the embryo, much less is known about morphogenesis and differentiation of these cells during post-embryonic stages. Previous studies of zebrafish revealed genetically distinct populations of embryonic and adult melanophores, the ectotherm homologue of amniote melanocytes. Here, we use molecular markers, vital labeling, time-lapse imaging, mutational analyses, and transgenesis to identify peripheral nerves as a niche for precursors to adult melanophores that subsequently migrate to the skin to form the adult pigment pattern. We further identify genetic requirements for establishing, maintaining, and recruiting precursors to the adult melanophore lineage and demonstrate novel compensatory behaviors during pattern regulation in mutant backgrounds. Finally, we show that distinct populations of latent precursors having differential regenerative capabilities persist into the adult. These findings provide a foundation for future studies of post-embryonic pigment cell precursors in development, evolution, and neoplasia.

## Author Summary

Understanding the biology of post-embryonic stem and progenitor cells is of both basic and translational importance. To identify mechanisms by which stem and progenitor cells are established, maintained, and recruited to particular fates, we are using the zebrafish adult pigment pattern. Previous work showed that embryonic and adult pigment cells have different genetic requirements, but little is known about the molecular or proliferative phenotypes of precursors to adult pigment cells or where these precursors reside during post-embryonic development. We show here that post-embryonic pigment cell precursors are associated with peripheral nerves and that these cells migrate to the skin during the larval-to-adult transformation when the adult pigment pattern forms. We also define morphogenetic and differentiative roles for several genes in promoting these events. Finally, we demonstrate that latent precursor pools persist into the adult and that different pools have different capacities for supplying new pigment cells in the context of pattern regeneration. Our study sets the stage for future analyses to identify additional common and essential features of pigment stem cell biology.

## Introduction

A fundamental challenge for modern developmental biology is to determine how populations of stem and progenitor cells are established, maintained, and recruited to differentiate at particular times and places during post-embryonic development and in the adult organism. The significance of the problem cannot be overstated. Not only are these cells essential for normal development and homeostasis, but understanding their biology has profound translational importance. If we seek to evoke regenerative responses in a clinical content, then post-embryonic stem and progenitor populations may well supply the cells for doing so [1]–[3]. If we hope to delay natural tissue senescence, it is the life cycle of these cells that may need to be manipulated [4]–[7]. And if we aim to control malignancy, these cells or their transformed progeny will often be our targets of choice [8]–[10].

Pigment cells are of great utility for understanding the biology of post-embryonic stem and progenitor cells. Pigment cells are a classic and enduring system for studying morphogenesis and differentiation, and a century of effort has provided a firm understanding of many aspects of pigment cell development in the embryo [11]–[14]. These cells arise from neural crest cells, which migrate from the dorsal neural tube and contribute not only to pigment cells, but also glia and neurons of the peripheral nervous system, bone and cartilage of the craniofacial skeleton, and more. Despite the long-standing interest in these embryonic events, it is now clear that pigment cells of adults derive in large part from post-embryonic stem cells that are themselves of neural crest origin [15]–[18]. We know some of the mechanisms that underlie post-embryonic precursor development yet many outstanding questions remain. Foremost among these concern the genes and cellular behaviors by which pigment stem or progenitor cells are established during early development and subsequently maintained, whether there exist distinct subpopulations of such cells with different genetic requirements and potentials, and how these cells are recruited during normal development and homeostasis.

Answers to these questions will provide insights into the basic biology of the adult pigment cell lineage, and can inform our understanding of post-embryonic neural crest derivatives as well as stem and progenitor cells more generally. These answers are also of enormous biomedical significance, as the skin pigment cell of mammals, the melanocyte, is associated with a variety of human pathologies [19] and transformed cells of this lineage give rise to melanoma, one of the most common cancers [20], [21] and also one of the most deadly [21]–[25]. Poor outcomes reflect the inefficacy of non-surgical treatments and the highly invasive character of melanoma cells [26]–[29]. This invasiveness results in part from neural crest and melanocyte-specific factors that are already expressed by untransformed precursors, as well as lineage-specific factors that are re-expressed upon transformation [30]. Better understanding the genetic requirements and dynamics of melanocyte development and homeostasis can thus provide insights into the behaviors of transformed cells, and may suggest novel strategies for clinical intervention.

In recent years, the zebrafish has proven to be a tractable system for elucidating features of pigment cell development in the embryo and during the larval-to-adult transformation, a period of post-embryonic development analogous to later organogenesis, fetal and neonatal development of mammals [31]–[33]. In the embryo, neural crest cells differentiate into embryonic/early larval melanophores, the zebrafish homologue of mammalian melanocytes. Melanophores and melanocytes depend on many of the same genes and pathways [12], [14], [34], and melanomas with characteristics equivalent to those of human melanomas can be induced in zebrafish [18], [34], [35].

The development of zebrafish adult pigmentation involves a “pigment pattern metamorphosis” in which an embryonic/early larval pigment pattern is transformed into that of the adult [12], [33], [36]–[38]. Whereas the embryonic/early larval pigment cells and pattern develop by 3.6 SSL (standardized standard length [33]; about 4 days post-fertilization), new “metamorphic” melanophores begin to differentiate scattered over the flank by 5.9 SSL and ultimately migrate to form the adult stripes. Simultaneously, additional metamorphic melanophores appear already at sites of stripe formation, and many embryonic/early larval melanophores are lost. These events culminate in a juvenile pigment pattern by 11.0 SSL (4–5 weeks post-fertilization), consisting of two melanophore stripes bounding a lighter “interstripe”. Melanophores comprising these stripes reside in the “hypodermis” between the epidermis and the myotomes [39], [40]. Other adult melanophores are found in the epidermis, the dorsal scales, and the fins. Two additional classes of pigment cells also develop: yellow–orange xanthophores, which populate the interstripe and are required for organizing melanophores into stripes [38], [41], [42]; and iridescent iridophores, which are initially limited to the interstripe but later occupy melanophore stripes as well [33]. During later adult development, additional stripes and interstripes are added as the fish grows.

Mutants with pigment pattern defects limited to post-embryonic stages have suggested a model in which embryonic/early larval melanophores develop directly from the neural crest, whereas metamorphic melanophores develop from latent stem cells of presumptive neural crest origin. For example, *picasso* and *puma* mutants have normal embryonic/early larval melanophores, but profound deficiencies in their complements of metamorphic melanophores. *picasso* encodes the neuregulin receptor erbb3b, which acts both autonomously and non-autonomously to the metamorphic melanophore lineage. Pharmacological inhibition of ErbB signaling further revealed that *erbb3b* activity is required during neural crest migration for the later development of metamorphic melanophores, suggesting this locus is essential for establishing a pool of precursors that will differentiate only later during the larval-to-adult transformation [43]. By contrast, *puma* encodes tubulin alpha 8-like 3a (tuba8l3a) and acts autonomously to the metamorphic melanophore lineage. The temperature sensitivity of this allele allowed the identification of a critical period during pigment pattern metamorphosis, suggesting a role in maintaining or expanding a population of latent precursors, or recruiting these cells as melanophores [36], [44], [45].

To date it has not been known where latent precursors to metamorphic melanophores reside, how *erbb3b*, *tuba8l3a* or other loci promote the normal morphogenesis and differentiation of these cells and their progeny, or whether pigment cell precursors have indefinite or more limited re-population potential. Here we investigate these issues using molecular marker analyses, transgenesis, vital labeling, and time-lapse imaging in wild-type and mutant backgrounds. We show that during post-embryonic development, proliferative pigment cell precursors are associated with peripheral nerves and ganglia, and migrate to the hypodermis during pigment pattern metamorphosis where they differentiate as melanophores and iridophores. Nerve-associated pigment cell precursors are missing or reduced in ErbB-deficient and *tuba8l3a* mutant backgrounds. By contrast, these precursors persist in other mutants having less severe metamorphic melanophore deficiencies, though their subsequent development is marked both by defects, and partial regulation, of morphogenesis and differentiation. Finally, we show that latent precursors persist into the adult but that different precursor pools have different regenerative potentials. These findings provide a critical context for understanding the cellular bases of adult melanophore development, the mechanistic underpinnings of mutant phenotypes, and the roles for latent precursors in adult homeostasis, regeneration, and neoplasia.

## Results

### A melanogenic, *erbb3b*-dependent population of extra-hypodermal progenitor cells

**Figure 1:**
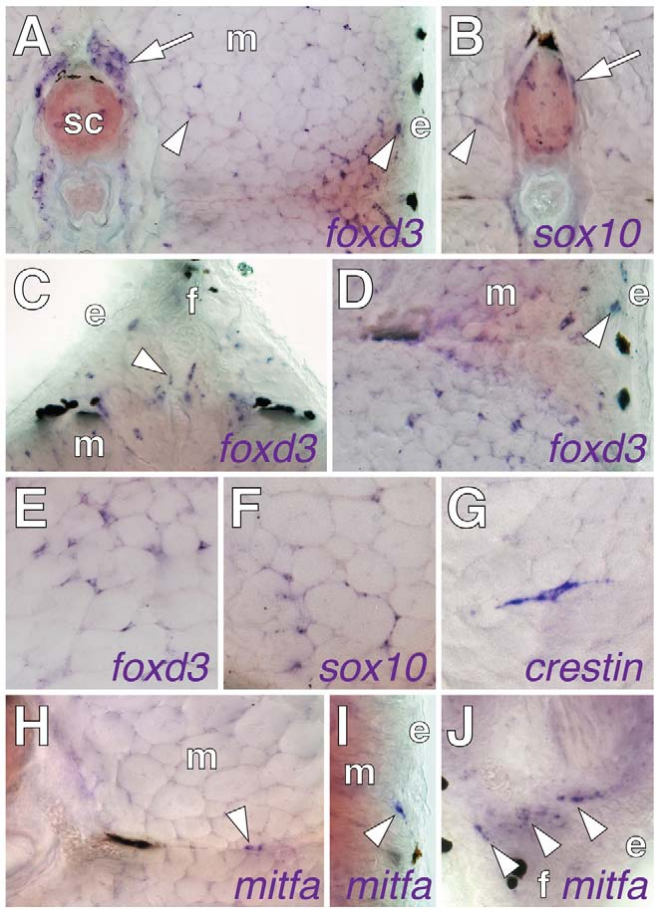
Post-embryonic expression of embryonic neural crest and glial markers. Shown are in situ hybridizations performed on transverse sections of 7–9 SSL larvae. (A) *foxd3* transcript was detected in dorsal root ganglia (arrow) and scattered cells (e.g., arrowheads) within the myotome (m) and near the epidermis (e). (B) *sox10* expression by cells adjacent to the neural tube (arrow) and within the myotome (arrowhead). (C) *foxd3+* cells (e.g., arrowheads) at the base of the dorsal fin (f). (D) *foxd3+* cells within the myotomes and near the epidermis. (E–G) *foxd3+*, *sox10+*, and *crestin+* cells within the myotomes. (H–J) Cells expressed *mitfa* (arrowheads), within the horizontal myoseptum (H), at the surface of the myotome (I), and at the base of anal fin (J) (see text for details).

To identify tissues that might harbor latent precursors to adult melanophores, we examined post-embryonic zebrafish for transcripts expressed by embryonic neural crest cells, reasoning that some of the cells expressing such markers might comprise a population of undifferentiated melanophore precursors. We examined *foxd3* and *sox10*, which are expressed by early neural crest cells, subpopulations of neural crest-derived glia, and some other cell types [46]–[49], as well as *crestin*, which is known only to be expressed by neural crest cells and their derivatives [50]. Cells expressing these loci were present in the hypodermis where the adult pigment pattern forms, but also in the myotomes, adjacent to the spinal cord, and at the bases of the fins (Figure 1A–1G), raising the possibility of both hypodermal and “extra-hypodermal” precursors for metamorphic melanophores.

**Figure 2:**
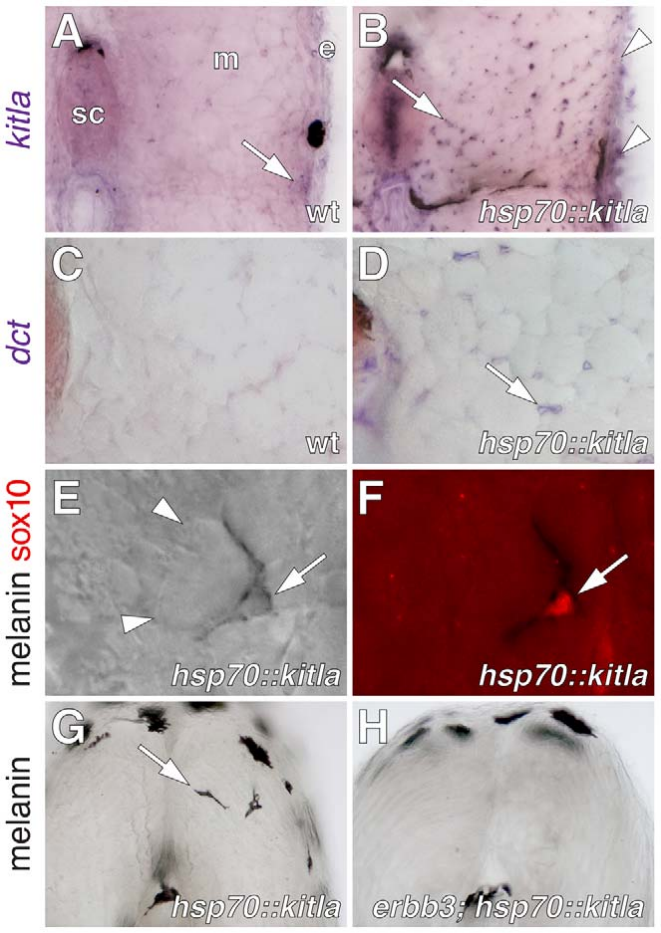
kitla misexpression induced ectopic melanophores within the myotomes. (A) *kitla* was normally expressed within the epidermis and hypodermis (arrow) during post-embryonic development of wild-type fish. sc, spinal cord. m, myotome, e, epidermis. (B) In sibling *Tg(hsp70::kitla)* larvae, heat shock resulted in increased *kitla* transgene expression within the epidermis (arrowheads) as well as ectopic expression within the myotomes (arrow), spinal cord, and elsewhere. (C) The late melanophore lineage marker *dopachrome tautomerase* (*dct*), encoding an enzyme required for melanin synthesis [96], [97], was not expressed within the myotomes of wild-type fish. (D) However, *dct* was expressed by scattered cells within the myotomes (arrow) in larvae misexpressing *kitla*. (E,F) Newly differentiated ectopic melanophores (arrow) were found between myotubes (arrowheads; E) and these cells continued to express sox10 protein (F). (G) Vibratome section revealing ectopic melanophores (arrow) within the myotome of a *Tg(hsp70::kitla)* larva 48 h after the initiation of kitla misexpression. Melanophores deep within the myotome were found only in *Tg(hsp70::kitla)*, though melanophores were occasionally found within the horizontal myoseptum of both transgenic and wild-type larvae [ectopic melanophores per larva, *Tg(hsp70::kitla)*: mean±SE = 1.3±0.15, range = 0–7 cells, *n* = 80 larvae; non-transgenic siblings: mean±SE = 0±0, range = 0, *n* = 69]. Longer durations of kitla misexpression resulted in more ectopic melanophores per larva. Suggesting that ectopic melanophores differentiated *in situ* rather than migrated into the myotomes from the hypodermis, labeling of hypodermal cells by photoconversion of mitfa::Eos+ [55] failed to reveal movement of cells away from enhanced kitla expression in the epidermis into the myotome (*n* = 10 larvae, 3–5 cells per individual). (H) In contrast to the wild-type, ectopic melanophores were significantly fewer in *erbb3b; Tg(hsp70::kitla)* mutants [Wilcoxon test, Z = 7.1, *P*<0.0001; ectopic melanophores per larva, *erbb3b; Tg(hsp70::kitla*): mean±SE = 0.04±0.03, range = 0–1, *n* = 50 larvae; non-transgenic siblings: mean±SE = 0±0, range = 0, *n* = 70] and in *Tg(hsp70::kitla*) larvae treated with AG1478 during the ErbB embryonic critical period [Wilcoxon test, Z = 2.9, *P*<0.005; ectopic melanophores per larva, AG1478-treated *Tg(hsp70::kitla*): mean±SE = 0.7±0.2, range = 0–4, *n* = 45 larvae; untreated *Tg(hsp70::kitla*) siblings: mean±SE = 1.6±0.2, range = 0–5, *n* = 35].

If extra-hypodermal, post-embryonic cells expressing genes typical of early neural crest cells contribute to metamorphic melanophores, we hypothesized that some of these cells should differentiate if supplied with appropriate trophic support. To test this idea, we used a heat-shock inducible transgenic line, *Tg(hsp70::kitla)^wp.r.t2^*, to misexpress the melanogenic factor kit ligand-a (kitla) [51] throughout the larva (Figure 2A, 2B). These larvae developed ectopic melanophores deep within the myotomes, which were never found in identically treated siblings lacking the transgene (Figure 2C–2G).

Previously we showed that *erbb3b* is essential for establishing precursors to metamorphic melanophores [43]. Accordingly, if extra-hypodermal kitla-responsive melanogenic cells and metamorphic melanophores arise from a common precursor pool, then ectopic melanophores should fail to develop in *erbb3b* mutants misexpressing kitla. Indeed, *erbb3b; Tg(hsp70:kitla)* larvae exhibited ∼30-fold fewer ectopic melanophores than wild-type *Tg(hsp70::kitla)* larvae (Figure 2H). A similar outcome was observed when wild-type larvae were treated with the ErbB inhibitor, AG1478, during the embryonic critical period for *erbb3b* activity in establishing metamorphic melanophore precursors [43]. Together, these results indicated that some extra-hypodermal cells are competent to differentiate as melanophores in the wild-type, but that most of these cells are missing when *erbb3b* activity is lost.

### Extra-hypodermal melanophore precursors are nerve-associated, proliferative, and specified for the melanophore lineage during the larval-to-adult transformation

Our identification of extra-hypodermal cells expressing genes typical of early neural crest and glial cells, as well as kitla-responsive melanogenic cells within the myotomes, led us to ask whether any of these cells embark upon a melanophore differentiation program during normal post-embryonic development. Since precursors to metamorphic melanophores require *erbb3b*, we further predicted that any candidate extra-hypodermal precursors of these cells should be missing in larvae deficient for *erbb3b* activity.

**Figure 3:**
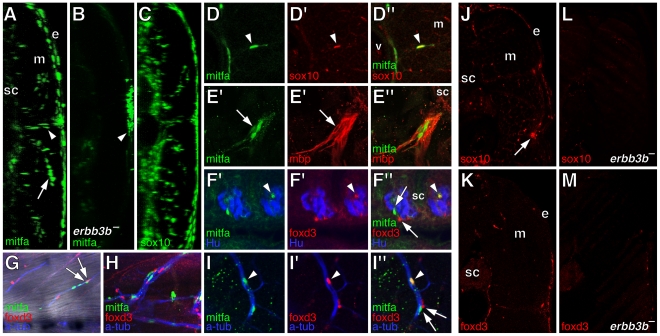
Extra-hypodermal cells expressing mitfa::GFP, foxd3, and sox10 in wild-type larvae and their deficiency in *erbb3b* mutants. All images are from early metamorphic (6.2–8.0 SSL) wild-type larvae except for B, L, and M, from *erbb3b* mutant larvae. (A–C) Transverse confocal projections (collapsing ∼1.5 mm of trunk along the anterior-posterior axis) showing GFP+ cells in living larvae (left side of each larva is shown). Images correspond to Videos S1, S2, S3. (A) mitfa::GFP+ cells in a wild-type fish occur in the hypodermis, between the epidermis (e) and the myotome (m), within the the myotome itself (arrow), and above the spinal cord (sc). Arrowhead, location of the horizontal myoseptum. (B) In *erbb3b* mutants, most mitfa::GFP+ cells were missing. This image is intentionally overexposed compared to A, revealing faint reflected fluorescence from iridescent iridophores in the hypodermis (arrowhead), which are present in wild-type larvae as well. (C) sox10::GFP+ cells were found in extra-hypodermal locations of wild-type *Tg(−4.9sox10:egfp)^ba2^* larvae [98]. (D–M) Immunohistochemical analyses of fixed specimens. (D) Co-expression of mitfa::GFP (green) and sox10 (red). v, vertebral column. (E) mitfa::GFP+ cells aligned with mbp+ glia (red) along ventral root motor fibers. Arrow, mitfa::GFP+ cells did not co-express mbp. (F) Lateral view showing mitfa::GFP+ cells and foxd3+ cells (red) between Hu+ neurons (blue) of dorsal root ganglia. mitfa::GFP+ and foxd3+ cells were often found close to one another (e.g., arrows) whereas other cells co-expressed mitfa::GFP and foxd3 (arrowhead). (G) Lateral view with superimposed brightfield and fluorescence images showing mitfa::GFP+ and foxd3+ cells along a peripheral nerve fiber stained for acetylated alpha tubulin (blue) within the myotome. Arrows, adjacent mitfa::GFP+ and foxd3+ cells. (H). A nerve plexus near the base of the caudal fin harbored numerous mitfa::GFP+ and foxd3+ cells. (I) Along a peripheral nerve within the myotome some cells co-expressed mitfa::GFP and foxd3 (arrowhead), whereas cells expressing either mitfa::GFP+ or foxd3+ were often juxtaposed (arrows). (J,K) Transverse sections through the dorsal trunk showing sox10+ cells (J) and foxd3+ cells (K) n the hypodermis, within the myotomes, and near the spinal cord. Arrow, lateral line nerve. (L,M) In *erbb3b* mutant larvae, very few sox10+ (L) or foxd3+ (M) cells were found.

In the wild-type, we found extra-hypodermal cells expressing *mitfa*, encoding a master regulator of melanophore fate specification (Figure 1H–1J) [52], [53]. Cells expressing *mitfa* in extra-hypodermal locations typically did so at lower levels than cells within the hypodermis and were at the limit of detection given the sensitivity of in situ hybridization at post-embryonic stages. However, we also identified extra-hypodermal cells expressing GFP driven by the proximal *mitfa* promoter in the transgenic line, *Tg(mitfa::GFP)^w47^*, which faithfully recapitulates *mitfa* expression in the melanophore lineage and in bipotent precursors to melanophores and iridophores in the embryo [54], [55] (Figure 3A; Videos S1, S2). In contrast to the wild-type, mitfa::GFP+ cells were largely absent from both *erbb3b* mutants and wild-type larvae treated with AG1478 during the embryonic *erbb3b* critical period (Figure 3B and see below; Video S3). In neither genetic background could we detect extra-hypodermal cells expressing transcript for *dct*, encoding a melanin synthesis enzyme.

The many extra-hypodermal mitfa::GFP+ cells in larvae contrasts with embryogenesis [54] and suggests that extra-hypodermal cells may be specified for the melanophore lineage during the larval-to-adult transformation. If so, we predicted that cells expressing markers typical of early neural crest cells (or glia) should occassionally be found to express mitfa::GFP as well. We therefore examined larvae for simultaneous expression of mitfa::GFP, sox10, and foxd3, and, to learn where these cells reside, we examined larvae with cell-type specific markers for surrounding tissues. These analyses revealed numerous mitfa::GFP+ cells associated with peripheral nerves and ganglia, including the doral root ganglia, ventral motor root fibers, lateral line nerve, and nerve fibers coursing through the myotomes (Figure 3E–3H; Figure S1). Ectopic melanophores within the myotomes of wild-type *Tg(hsp70:kitla)* larvae were likewise nerve-associated (Figure S2).

**Figure 4:**
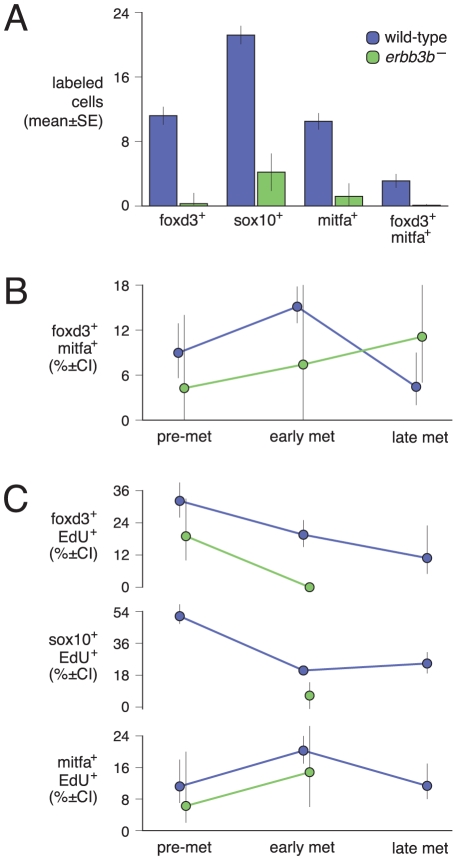
Missing extra-hypodermal precursor cells in *erbb3b* mutants, co-expression of molecular markers, and temporally regulated proliferation. (A) Occurrence of cells in sections from the mid-trunk of wild-type and *erbb3b* mutant larvae. Each class of cells was reduced in *erbb3b* mutants (all *P*<0.0001). (B,C) Cell frequencies in wild-type and *erbb3b* mutants across stages. pre-met, pre-metamorphosis (4.9–5.3 SSL); early met, early metamorphosis (6.2–8.0 SSL); late met, late metamorphosis (9.0–13.0 SSL). (B) The frequency of foxd3+; mitfa::GFP+ cells was greatest in wild-type larvae during early pigment pattern metamorphosis (doubly vs. singly labeled cells, difference among stages: *χ* *^2^* = 15.7, d.f. = 2, *P*<0.0005; *N* = 1217 total cells examined). Doubly labeled cells tended to be rarer and delayed in *erbb3b* mutants (*N* = 83 total cells examined). (C) The frequencies of EdU+ cells differed significantly among stages, with more foxd3+ and sox10+ cells labeled with EdU during the pre-metamorphic period, and more mitfa::GFP+ cells labeled with EdU during early metamorphosis (EdU labeling frequency variation among stages, foxd3+: *χ* *^2^* = 11.3, d.f. = 2, *P*<0.005, *N* = 450 cells; sox10+: *χ* *^2^* = 140.7, d.f. = 2, *P*<0.0001; *N* = 1679 cells; mitfa::GFP+: *χ* *^2^* = 14.4, d.f. = 2, *P*<0.001, *N* = 927 cells). In *erbb3b* mutants, EdU labeling frequencies were reduced in comparison to wild-type for foxd3+ cells (*χ* *^2^* = 3.4, d.f. = 1, *P* = 0.06; *N* = 44 cells) and sox10+ cells (*χ* *^2^* = 11.4, d.f. = 1, *P*<0.001; *N* = 77 cells), though not significantly so for mitfa::GFP+ cells (*P*>0.1; *N* = 59 cells). Asymmetric confidence intervals in B and C, Bayesian 95% upper and lower bounds.

Double label analyses with markers of neural crest, glial, and melanophore lineages revealed that, in wild-type larvae, mitfa::GFP+ cells were often in close proximity to cells expressing sox10 and foxd3, and also co-expressed sox10, as expected given the direct regulation of *mitfa* by sox10 (Figure 3D, 3G, 3H) [56], [57]. We also found that 4–15% of mitfa::GFP+ cells co-expressed foxd3, with doubly labeled cells occurring most frequently during early metamorphosis, when the rate of melanophore population increase is maximal [36] (Figure 3F, 3I; Figure 4A, 4B). This frequency of double labeling is reminiscent of 15–18 h embryos, in which 9–12% of mitfa::GFP+ cells are foxd3+ [54]. In contrast to the co-labeling of mitfa::GFP and foxd3, we did not find mitfa::GFP expression by myelinating glia expressing myelin basic protein (mbp) (Figure 3E; Figure S1). As anticipated, however, some cells expressing foxd3 or sox10 co-expressed mbp. All of these cell types were deficient in *erbb3b* mutants and wild-type larvae treated with AG1478 during the embryonic *erbb3b* critical period (Figure 3J–3M; Figure 4A, 4B; Figure S4), though a few residual mitfa::GFP+ and foxd3+ cells occurred in anterior and posterior regions, corresponding to axial levels where a few residual melanophores develop during metamorphosis [43] (data not shown).

**Figure 5:**
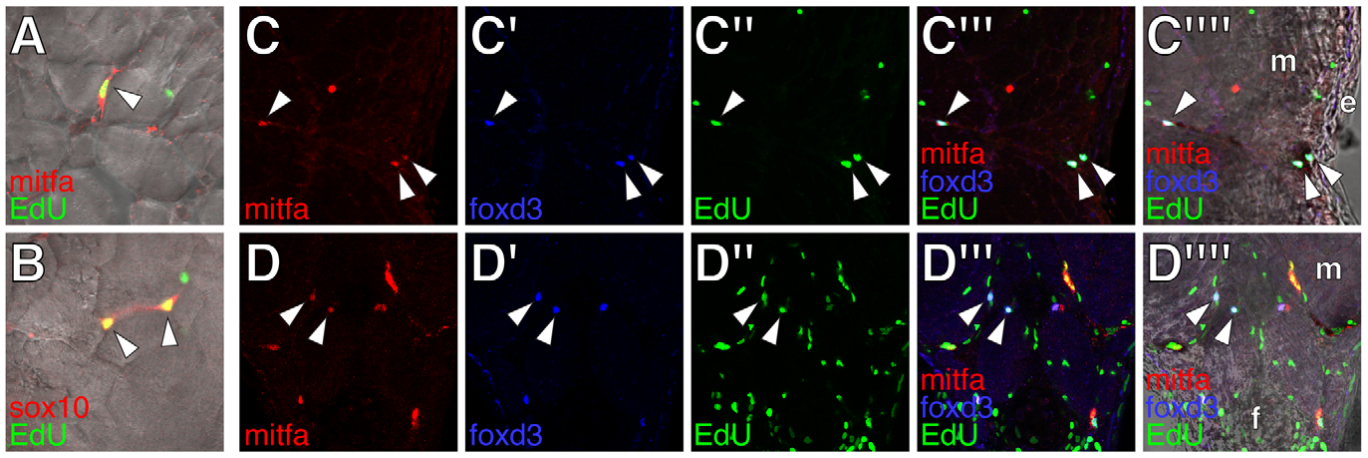
Proliferative extra-hypodermal cells revealed by post-embryonic EdU incorporation. All images from transverse sections of wild-type larvae staged as in Figure 3 (see Figure S4 for comparisons with *erbb3b* mutant larvae). (A,B) Merged images showing cells (arrowheads) within the myotomes labeled for either mitfa::GFP (red in A) or sox10::GFP (red in B) as well as EdU (green). (C,D) Cells within the lateral myotomes (m) and near the hypodermis (C) or at the base of the anal fin (f in D) labeled for mitfa::GFP (red), foxd3 (blue), and EdU (green). Merged views show fluorescence images or fluorescence images with brightfield overlays. Arrowheads, triple-labeled cells. m, myotome. e, epidermis.

If some foxd3+ and sox10+ cells are progenitors to post-embryonic melanoblasts, we predicted that a period of population expansion could precede the appearance of mitfa::GFP+ cells, which might themselves be proliferative. Consistent with this idea, we found EdU incorporation by all three cell types but EdU-labeling of post-embryonic foxd3+ and sox10+ cells was most frequent prior to pigment pattern metamorphosis, whereas EdU-labeling of mitfa::GFP+ cells was most frequent during the peak of pigment pattern metamorphosis (Figure 4C, Figure 5). Given these findings, we further asked if doubly labeled foxd3+; mitfa::GFP+ cells constitute an especially proliferative population. These analyses revealed EdU-incorporation in 55% of foxd3+; mitfa::GFP+ cells, a significantly higher frequency than for cells expressing only foxd3 (17% EdU+) or only mitfa::GFP (24% EdU+; *χ* *^2^* = 131.7, d.f. = 2, *P*<0.0001). The frequency of EdU labeling amongst foxd3+; mitfa::GFP+ cells did not vary significantly across stages (*χ* *^2^* = 2.3, d.f. = 2, *P* = 0.3). Finally, in *erbb3b* mutants sampled at selected stages, we found lower levels of EdU incorporation than in wild-type, though small numbers of cells overall resulted in correspondingly low statistical power (Figure 4C; Figure S4).

The foregoing analyses indicated that, during post-embryonic development, a proliferative population of extra-hypodermal, *erbb3b*-dependent foxd3+ and sox10+ cells associated with peripheral nerves and ganglia becomes specified as precursors to melanophores (or as bipotential precursors to melanophores and iridophores; see reference [55]).

### Extra-hypodermal cells contribute to the hypodermal populations of metamorphic melanophores and iridophores

**Figure 6:**
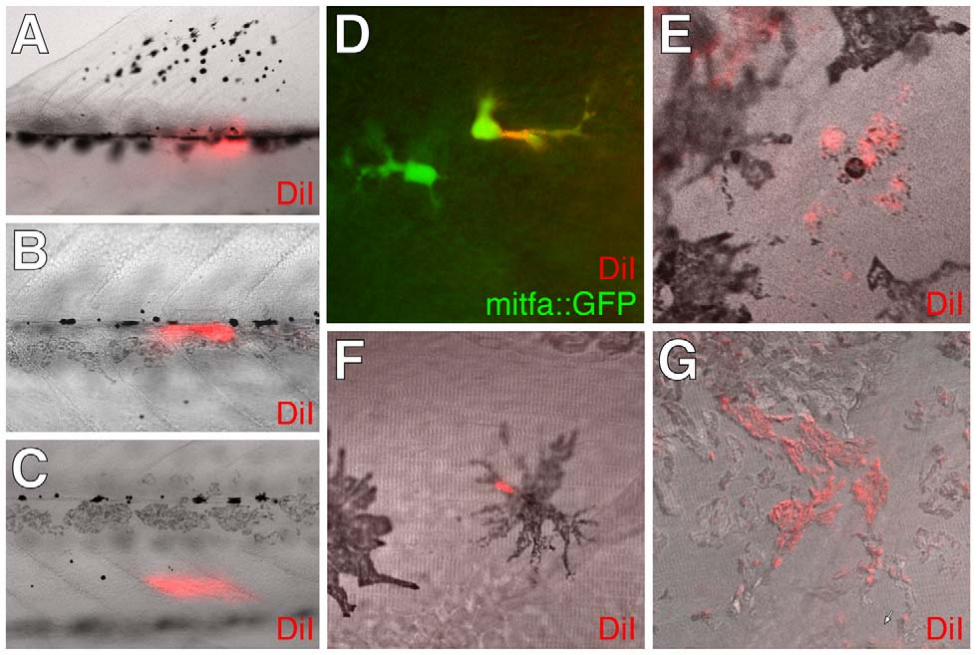
DiI-labeling showed extra-hypodermal contributions to metamorphic melanophores and iridophores. (A–C) DiI labeled tissues imaged immediately after injection into the base of the dorsal fin (A), the vicinity of the horizontal myoseptum and lateral line nerve (B), and the inner myotome (C). Each site yielded hypodermal DiI+; mitfa::GFP+ cells or DiI+ melanophores (12 of 30 larvae, 3 of 30 larvae, 15 of 87 larvae, respectively). (D–F) DiI+ cells that expressed either mitfa::GFP (D) or contained melanin (E,F) found within the lateral hypodermis 4 d following injection into the base of the dorsal fin (D, F) or the inner myotome (E). (G) DiI-labeling was observed for additional cells including iridophores. Although the frequencies with which DiI labeled pigment cells were found differed between injection sites, each site gave rise to DiI+ iridophores at a frequency indistinguishable from that of DiI+ mitfa::GFP+ cells (*χ* *^2^* = 0.6, d.f. = 1, *P* = 0.4). We did not observe DiI-labeled xanthophores.

The development of post-embryonic, extra-hypodermal mitfa::GFP+ cells suggested the possibility that such cells migrate to the hypodermis during metamorphosis. To test this idea, we injected DiI into the myotomes, the horizontal myoseptum, or the base of the dorsal fin of wild-type or *Tg(mitfa::GFP)* larvae and we assessed after ≥4 d whether DiI-labeled cells were present within the hypodermis distant from the injection sites. In 10–30% of injected larvae we found hypodermal DiI-labeled melanized cells, mitfa::GFP+ cells, or iridophores (Figure 6), indicating that some extra-hypodermal cells migrated to the hypodermis during metamorphosis.

**Figure 7:**
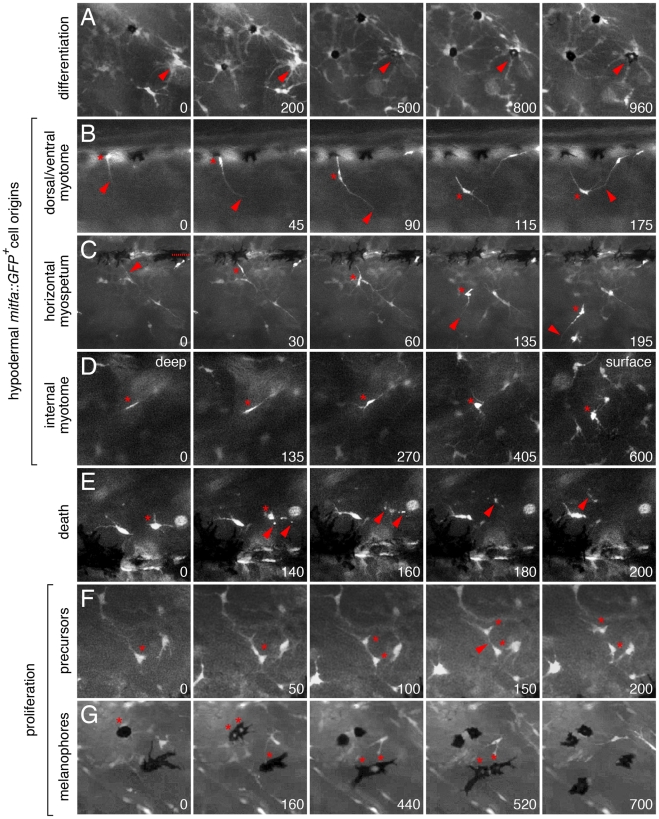
*Ex vivo* time-lapse imaging revealed extra-hypodermal origins and morphogenetic behaviors of hypodermal mitfa::GFP+ cells and melanophores. All panels show lateral views of larval trunks and are derived from time-lapse movies of *Tg(mitfa::GFP)* larvae. Elapsed time (min) at lower right of each panel. (A) mitfa::GFP+ cells differentiated into melanophores (e.g., arrowhead). (B–D) mitfa::GFP+ cells entered the hypodermis during the larval-to-adult transformation. (B) A cell at the dorsal margin of the myotomes extended a long process (arrowhead) into the hypodermis and interacted with processes of a second cell. *, cell body. (C) A long process (arrowhead) preceded emergence of the cell body (*) from the level of the horizontal myoseptum (dotted line). This cell subsequently interacted with a neighboring cell, extended a processes ventrally, and moved in that direction. (D) A cell initially deep within the myotome (*) emerged into the hypodermis. The focal plane changes across panels, from deep within the myotome to the surface of the myotome and hypodermis, where other cells are found already. (E) Death of mitfa::GFP+ cell (*) revealed by fragmentation and cellular debris (arrowheads). (F,G) mitfa::GFP+ cells (F) and melanophores that retain some residual GFP expression (G) proliferating within the hypodermis. Melanophores in G are imaged in a *kita* mutant (see text for details). See Videos S4, S5, S6, S7, S8, S9, S10, S11, S12, S13, S14, S15, S16.

**Figure 8:**
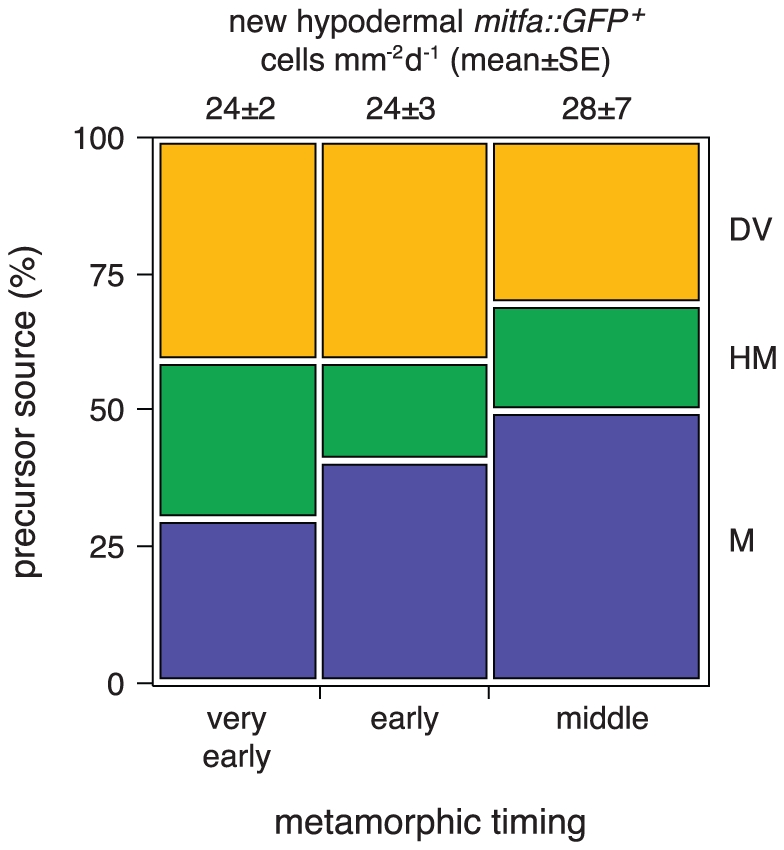
Sources of mitfa::GFP+ cells that entered the hypodermis. Shown are the relative frequencies of cells entering the hypodermis in time-lapse movies of trunks derived from wild-type larvae (*N* = 127 larvae, 1220 mitfa::GFP+ total cells examined) during different periods of pigment pattern metamorphosis (very early, 6.4–6.6 SSL; early, 6.6–6.8 SSL; middle, 6.8–7.6 SSL). Cells newly arrived in the hypodermis (*n* = 644) were classified as migrating over the dorsal or ventral margins of the myotomes (DV), emerging from the vicinity of the horizontal myoseptum (HM) and lateral line, or emerging from within the myotomes (M), tyically along vertical myosepta. Individuals were binned by stage and bar widths are proportional to the average numbers of newly appearing hypodermal mitfa::GFP+ cells per larva, normalized to cells mm^−2^ day^−1^ (mean±SE above bars). The relative frequencies of cells arising from different sources differed between stages (*χ* *^2^* = 20.0, d.f. = 4, *P*<0.0001), with cells increasingly likely to emerge from within the myotomes as compared to migrating over the dorsal or ventral margins of the myotomes.

To further test the hypothesis that extra-hypodermal cells contribute to the hypodermal melanophore population, we examined cell behaviors by time-lapse imaging of trunks derived from *Tg(mitfa::GFP)* larvae. Movies revealed the differentiation of mitfa::GFP+ cells into melanophores as well as their migration (Figure 7A; Videos S4, S5, S6). We therefore assessed the migratory pathways by which mitfa::GFP+ cells had reached the hypodermis. Approximately half of all mitfa::EGFP+ cells arrived within the hypodermis during imaging. Some entered the hypodermis after migrating over the dorsal or ventral margins of the myotomes, whereas others originated from within the myotomes, emerging either from the vicinity of the horizontal myoseptum or along vertical myosepta (Figure 7B–7D, Figure 8; Videos S7, S8, S9, S10). Movies also revealed the movement of mitfa::GFP+ cells along nerves and their detachment from nerves to migrate more broadly through the fish (Videos S11, S12). Together then, DiI labeling and time-lapse imaging indicated that extra-hypodermal cells contribute to hypodermal mitfa::GFP+ cells, melanophores, and iridophores during the larval-to-adult transformation.

### Genetic requirements for extra-hypodermal precursor morphogenesis and differentiation

Our findings suggested that a normal complement of adult melanophores depends on contributions from a pool of extra-hypodermal precursors. If this is the case, we predicted that mutants with severe metamorphic melanophore deficiencies should have correspondingly severe deficiencies of extra-hypodermally derived mitfa:GFP+ cells. To test the contributions of extra-hypodermal cells to hypodermal mitfa::GFP+ cells and melanophores, we crossed the *Tg(mitfa::GFP)* transgene into *erbb3b* and *tuba8l3a* mutants, which exhibit severely reduced numbers of metamorphic melanophores [36], [43], [44].

**Figure 9:**
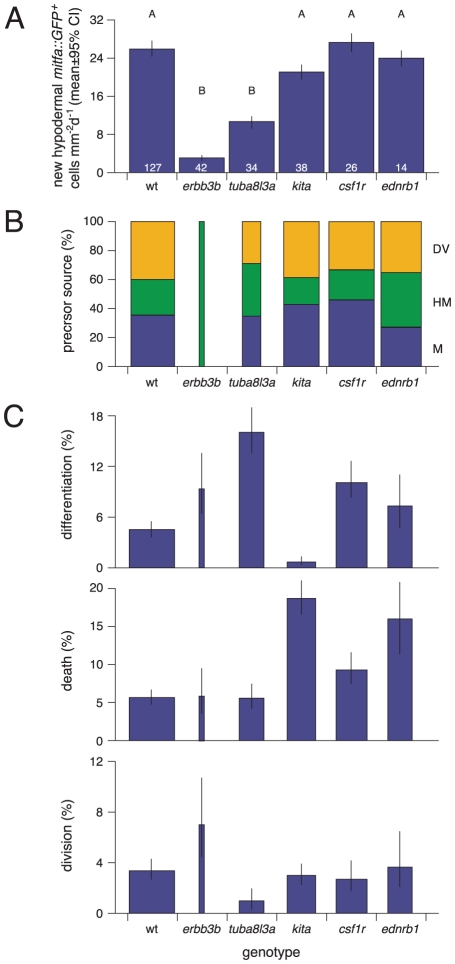
Genetic controls over the origins, differentiation, and morphogenesis of hypodermal mitfa::GFP+ cells. Shown are analyses of time-lapse movies for trunks derived from wild-type and mutant larvae (*N* = 281 larvae, 5241 total cells examined). (A) Total numbers of newly arising hypodermal mitfa::GFP+ cells differed among genotypes (square root transformed data, *F*_5,273_ = 30.2, *P*<0.0001). Shown are least squares means (±95% confidence intervals) after controlling for significant differences among stages (*F*_2,273_ = 3.9, *P*<0.0001) and normalized to cells mm^−2^ day^−1^. Letters above bars indicate means that differed significantly (*P*<0.05) by Tukey-Kramer post hoc comparisons. Numbers within bars indicate numbers of larval trunks examined. (B) The origins of new hypodermal mitfa::GFP+ cells differed among genotypes (*χ* *^2^* = 145.6, d.f. = 10, *P*<0.0001; *n* = 1582 total new cells). Bar widths are proportional to the total numbers of new hypodermal cells observed in each genotype (shown in A). DV, cells entering the hypodermis after migrating over the dorsal or ventral myotome margins; HM, cells entering from the vicinity of the horizontal myoseptum. M, cells entering from within the myotomes. The sources of mitfa::GFP+ cells did not differ significantly across stages overall (*χ* *^2^* = 0.003, d.f. = 4, *P* = 1), though different genotypes exhibited stage-dependent variation (stage x genotype interaction: *χ* *^2^* = 46.3, d.f. = 20, *P*<0.0001; not shown). (C) Frequencies of differentiation, death and proliferation differed among genotypes. Bar widths are proportional to the total numbers of hypodermal mitfa::GFP+ cells and melanophores observed per larva, after controlling for area and duration of imaging, and normalized to cells mm^−2^ day^−1^ (larva means±SE: wild-type, 116±9; *erbb3b*, 13±8; *tuba8l3a*, 62±9; *kita*, 72±10; *csf1r*, 78±14; *ednrb1*, 61±16). Differentiation, The likelihood of mitfa::GFP+ cells acquiring melanin during imaging differed among genotypes (*χ* *^2^* = 100.6, d.f. = 5, *P*<0.0001; *n* = 335 total differentiating cells): the relatively few *erbb3b* and *tuba8l3a* mutant cells were especially likely to differentiate whereas very few *kita* mutant cells differentiated. Additional effects were attributable to stage (*χ* *^2^* = 30.3, d.f. = 2, *P*<0.0001) and a stage x genotype interaction (*χ* *^2^* = 27.3, d.f. = 10, *P*<0.0001; not shown). Death, The incidence of mitfa::GFP+ cells dying during imaging differed among genotypes (*χ* *^2^* = 116.9, d.f. = 5, *P*<0.0001; *n* = 507 total dying cells) with particularly high rates of death in *kita* and *ednrb1* mutants. Additional variation was attributable to differences among stages (*χ* *^2^* = 23.5, d.f. = 2, *P*<0.0001) and a stage x genotype interaction (*χ* *^2^* = 20.4, d.f. = 10, *P*<0.05) resulting from an increased likelihood of *ednrb1* mutant cells dying at later stages (not shown) Division, The incidence of mitfa::GFP+ cells dividing differed significantly among genotypes (*χ* *^2^* = 23.6, d.f. = 5, *P*<0.0001; *n* = 142 total dividing cells). Asymmetric confidence intervals, Bayesian 95% upper and lower bounds.

**Figure 10:**
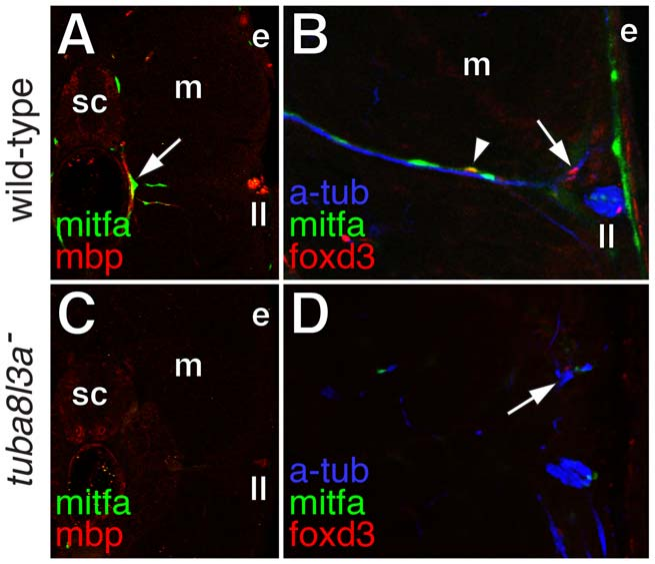
Extra-hypodermal precursors were deficient in *tuba8l3a* mutant larvae. (A) Wild-type larvae exhibited mitfa::GFP+ cells (green) associated with mbp+ glia (red) of peripheral nerves (arrow). sc, spinal cord; m, myotome; e, epidermis; ll, lateral line nerve. (B) mitfa::GFP+ cells (green), foxd3+ cells (red; arrow), and doubly labeled mitfa::GFP+; foxd3+ cells (arrowhead) were associated with nerve fibers stained for acetylated alpha tubulin (blue). (C) In *tuba8l3a* mutants, regions deficient for mbp+ glia were also deficient for mitfa::GFP+ cells. (D) Peripheral nerves were often defasciculated (arrow) and were deficient for foxd3+ and mitfa::GFP+ cells.

In comparison to the wild-type, and as predicted from the foregoing analyses, *erbb3b* mutants had dramatically fewer extra-hypodermally derived mitfa::GFP+ cells (Figure 9A; Video S13). *erbb3b* mutant mifa::GFP+ cells originated from the vicinity of the horizontal myoseptum (Figure 9B), and once in the hypodermis, these cells were more likely to differentiate and to divide (Figure 9C; in contrast to the somewhat reduced rates of EdU incorporation prior to reaching the hypodermis shown in Figure 4C). *tuba8l3a* mutants also had significantly fewer extra-hypodermally derived mitfa::GFP+ cells. These cells were more likely to differentiate, but divided at only one-third the frequency of the wild-type (Figure 9; Video S14). *tuba8l3a* mutants exhibit a post-embryonic demyelination of the peripheral nervous system [44], and we found that regions exhibiting mbp+ glial deficiencies and peripheral nerve defasciculation had fewer mitfa::GFP+ and foxd3+ cells (Figure 10). We did not observe cells doubly labeled for foxd3 and mitfa::GFP in *tuba8l3a* mutants.

Amongst the *erbb3b*- and *tuba8l3a*-dependent metamorphic melanophore populations, are temporally and genetically distinct subpopulations, comprising early metamorphic melanophores that are initially dispersed but later migrate into stripes, and late metamorphic melanophores that develop already at sites of stripe formation [37], [43], [45] (Figure S5). Early metamorphic melanophores are ablated in *kita* mutants, but persist in *colony stimulating factor-1 receptor* (*csf1r*) and *endothelin receptor b1* (*ednrb1*) mutants. By contrast, late metamorphic melanophores persist in *kita* mutants, but are ablated in *csf1r* and *ednrb1* mutants [37], [38], [58], [59]. To test if these differences reflect differential persistence of distinct precursor pools, or differences in the subsequent morphogenesis and differentiation of cells arising from a common precursor pool, we crossed the *Tg(mitfa::GFP)* transgene into *kita^b5^*, *csf1r^j4e1^* and *ednrb1^b140^* mutants and examined the origins of hypodermal mitfa::GFP+ cells as well as their frequencies of differentiation, death and proliferation.

In contrast to *erbb3b* and *tuba8l3a* mutants, *kita*, *csf1r*, and *ednrb1* mutants did not exhibit significantly fewer extra-hypodermally derived mitfa::GFP+ cells than the wild-type, though cells in *kita* mutants typically failed to differentiate and instead died at high frequency, whereas cells in *csf1r* and *ednrb1* mutants were more likely both to differentiate and to die (Figure 9; Video S15). We did not observe gross defects in mitfa:GFP+ cell motility in any of the mutant backgrounds. Finally, in contrast to the proliferation of unmelanized mitf::GFP+ cells (Figure 9C), proliferation of differentiated melanophores was rare in the wild-type (0.1%; *N* = 3822 melanophores observed) and in most of the mutants (0.2–0.4%; *N* = 4358 melanophore observed). In *kita* mutants, however, the few melanophores that differentiated divided frequently (14%; *N* = 35 melanophores observed; variation among genotypes: *χ* *^2^* = 38.1, d.f. = 5, *P*<0.0001; Video S16). Together these data show that e*rbb3b* and *tuba8l3a* each promote the development of extra-hypodermal mitfa::GFP+ cells, whereas all five loci promote the differentiation and morphogenesis of these cells once they reach the hypodermis.

### Latent melanophore precursors in adult zebrafish

**Figure 11:**
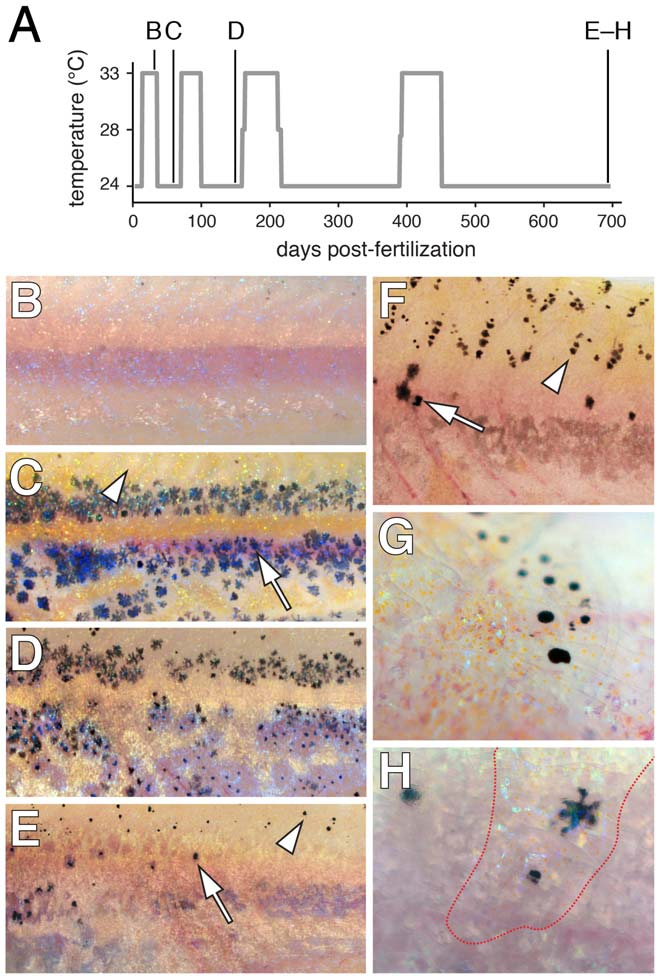
Limited regeneration of adult hypodermal melanophores following genetic ablation. (A) Time-course of temperature shifts, with letters corresponding to images in B–H. Final sample size at 698 days post-fertilization: *n* = 5. (B) A young adult *kita; csf1r^TS^* mutant at 33°C lacked melanophores as in *kita; csf1r^j4e1^* mutants [38] (some melanized cellular debris resulting from melanophore death is evident dorsally). (C) Temperature downshift to 24°C allowed recovery of a hypodermal melanophore (e.g., arrow) complement initially indistinguishable from *kita* single mutants [59]. Arrowhead, the dorsal flank is initially devoid of scale-associated melanophores, as is typical of *kita* mutants. (D,E) Additional rounds of ablation and recovery yield progressively fewer hypodermal melanophores (arrow), though some melanophores develop on the dorsal scales (arrowhead). Hypodermal xanthophores and iridophores were depleted as well (data not shown). (F) Dorsal flank of another individual showing hypodermal melanophores (arrow) and scale melanophores (arrowhead). (G) Detail of scale-associated melanophores. Iridescent iridophores and yellow-orange xanthophores are apparent as well. (H) Detail of hypodermal melanophores viewed through an overlying scale containing a concentration of xanthophores and iridophores (outlined in red).

Our demonstration that extra-hypodermal precursors contribute to hypodermal melanophores led us to ask whether latent pigment cell precursors persist into the adult. Extra-hypodermal foxd3+ and mitfa::GFP+ cells were distributed in adult fish similarly to metamorphic stages and also were found associated with the scales (Figure S6). To test the capacity of latent precursors to supply new melanophores, we sought to ablate melanophores with the goal of provoking a regenerative response. Because fish doubly mutant for *kita^b5^* and presumptive null alleles of *csf1r* lack body melanophores [38], we reasoned that fish doubly mutant for *kita^b5^* and the temperature-sensitive allele *csf1r^ut^* ^.*r1e174*^ (*csf1r^TS^*) [60] should have fewer melanophores (equivalent to *kita^b5^* single mutants) at permissive temperature, but should lack all melanophores at restrictive temperature. Repeated exposure to restrictive and permissive temperatures should thus allow for repeated cycles of ablation and regeneration of these *kita*-independent, *csf1r*-dependent melanophores. As predicted, *kita; csf1r^TS^* double mutants that were initially indistinguishable from *kita* single mutants lost body melanophores when shifted to restrictive temperature (Figure 11A, 11B). After returning to permissive temperature, fish initially recovered *kita*-independent hypodermal melanophores, though progressively fewer of these cells were regenerated in successive ablation–recovery cycles (Figure 11C, 11D). Surprisingly, ablations also resulted in the *de novo* development and regeneration of scale melanophores, which are normally absent from *kita* mutants (Figure 11F, 11G)[37]. The few later hypodermal melanophores that were recovered in *kita; csf1r^TS^* mutants were often located beneath scales populated with melanophores, iridophores and xanthophores (Figure 11H), raising the possibility that some of these regenerative hypodermal melanophores may have been scale-derived. Overall, these findings suggest that precursors to *kita*-independent, *csf1r*-dependent hypodermal melanophores persist in the adult yet have a finite regenerative potential, whereas an additional precursor pool associated with adult scales has a greater regenerative capability.

## Discussion

**Figure 12:**
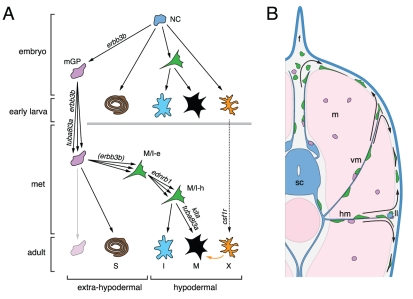
Model for establishment and maintenance of adult pigment cell precursors and their recruitment during development and regeneration. (A) Hypothesized lineage relationships, showing neural crest (NC) cells in the early embryo that give rise to Schwann cells and pigment cells of the early larva as well as *erbb3b*-dependent progenitors to metamorphic glial and pigment cell lineages (mGP). mGP are maintained in association with peripheral nerves and ganglia, express foxd3, and their population expands (multiple arrowheads) in a *tuba8l3a*-dependent manner. During pigment pattern metamorphosis (met), some mGP differentiate as metamorphic Schwann cells (S) and the expansion of this lineage likely requires *erbb3b* (not shown). Other mGP become specified for metamorphic pigment cell lineages, as marked by mitfa::GFP expression. Some mitfa::GFP+ cells will give rise to melanophores or iridophores and are initially extra-hypodermally located in peripheral nerves and ganglia (M/I-e) but then migrate to the hypodermis (M/I-h). The expansion of this population requires *ednrb1* [58]. Some M/I-h will differentiate as metamorphic iridophores (I), other M/I-h expand their population in a *tuba8l3a-* and *kita-*dependent manner and ultimately differentiate as metamorphic melanophores (M). Individual M/I-e or M/I-h may be bipotent for melanophore and iridophore fates, as in embryos [55], or their respective populations may harbor precursors already specified for either the melanophore or iridophore fate. *csf1r*-dependent metamorphic xanthophores (X) presumably arise from a different precursor population (dashed line) and promote the survival of metamorphic melanophores (orange arrow) [38], [42], [60]. Some mGP persist into the adult and have a limited re-population potential. (B) Schematic of metamorphic larva illustrating sources and migratory pathways of metamorphic melanophore and iridophore precursors. Shown are mGP and M/I-e (colors as in A) associated with nerves and beneath the dorsal fin (f). M/I-e enter the hypodermis (arrows) from the dorsal or ventral margins of the myotomes (m), or after migrating along nerves associated with the the vertical myosepta (vm) or the horizontal myoseptum (hm). Others may arise from the lateral line nerve (ll). Once in the hypodermis, these cells differentiate as melanophores (green cell with heavy black outline) or iridophores (not shown). sc, spinal cord. Additional populations of precursors that may give rise to LM melanophores and scale melanophores are not shown (see text).

The results of this study and previous analyses [36], [43], [45] suggest a model for the development of adult melanophores in zebrafish (Figure 12). Pluripotent foxd3+ precursors to glia [46], [61], adult melanophores and iridophores are established in an *erbb3b*-dependent manner during embryogenesis, and thereafter are associated with post-embryonic peripheral nerves and ganglia. This precursor population is expanded and maintained during pre-metamorphic larval development in an *erbb3b*- and *tuba8l3a*-dependent manner, and cells within this pool become specified for pigment cell lineages beginning immediately before, and continuing through, pigment pattern metamorphosis. During the larval-to-adult transformation, these extra-hypodermal precursors migrate to the hypodermis, and there contribute to metamorphic melanophores and iridophores. Some enter the hypodermis after migrating over the dorsal or ventral margins of the myotomes, others emerge from vertical or horizontal myosepta; some may emigrate from the lateral line nerve. Once in the hypodermis, these cells require *tuba8l3a* for their proliferation, as well as *kita*, *ednrb1*, and, to a lesser extent, *csf1r*, for their survival and eventual differentiation. Later in adults, some latent precursors persist and can supply a limited number of new hypodermal melanophores, whereas other precursors associated with scales have a greater regenerative capacity. Below we discuss several aspects and implications of this model.

### Extra-hypodermal niches and the *erbb3b*- and *tuba8l3a*-dependence of latent precursors to adult melanophores

A major finding of our study is that post-embryonic mitfa::GFP+ cells are associated with peripheral nerves coursing through the myotomes as well as more medial nerves and ganglia. We further showed that nerve-associated cells could be induced to differentiate ectopically as melanophores, and that extra-hypodermal mitfa::GFP+ cells migrate to the hypodermis where some differentiate as melanophores during normal development. These observations suggest that peripheral nerves or ganglia serve as niches for post-embryonic precursors to adult melanophores and are broadly consistent with a recent study demonstrating a peripheral nerve origin for adult skin melanocytes of amniotes [62] as well as analyses revealing interconversion of glial and melanocyte fates *in vitro* [63]–[65]. Our study complements and extends recent lineage tracing studies of flounder larvae, in which adult pigment cell precursors were found to migrate to the hypodermis from dorsal and ventral regions during pigment pattern metamorphosis [66], [67].

Our analyses also provide insights into the molecular and proliferative phenotypes of metamorphic melanophore precursors. foxd3 often acts as a transcriptional repressor and is associated with the maintenance of pluripotency and pluripotent cells [68]–[71]. In the neural crest lineage, foxd3 is expressed by pluripotent cells and presumptive glia, and can inhibit *mitfa* transcription, favoring iridophore or glial over melanogenic fates [54], [55], [61], [72]. We found that some nerve-associated foxd3+ cells co-expressed mitfa::GFP just before and continuing through pigment pattern metamorphosis, and that such cells were especially likely to have incorporated EdU. These observations raise the possibility that nerve-associated foxd3+ cells are a pluripotent and proliferative population that can give rise to hypodermal melanophores and iridophores during pigment pattern metamorphosis. Our finding that DiI-labeled, extra-hypodermal tissues give rise to melanophores and iridophores with equal frequency is consistent with this idea. Because the mitfa::GFP transgene we employed is repressible by foxd3, we speculate that co-expression of mitfa::GFP reflects low levels of perduring foxd3 protein as precursors adopt a pigmentary fate, similar to observations of early neural crest morphogenesis [54], or that a balance between the anti-melanogenic and pro-melanogenic effects of foxd3 and mitfa, respectively, prevents specified cells from differentiating prematurely. Nevertheless, we note that our analyses revealed more extra-hypodermal mitfa::GFP+ cells than *mitfa+* cells, which differs from the one-to-one corrrespondence of such cells in embryos [54]. Additional mitfa::GFP+ cells could reflect low levels of *mitfa* expression that fall below the threshold for detection by in situ hybridization at post-embryonic stages, yet are sufficient for accumulating detectable levels of relatively stable GFP. Or, mitfa::GFP expression in some cells could reflect a partial disregulation of the transgene, as might occur if regulatory elements for post-embryonic expression are missing. Distinguishing between these possibilities will require the production and analysis of additional transgenic reporter lines, but whichever the outcome, the mitfa::GFP reporter we have used will be a valuable tool for further dissecting the mechanisms of post-embryonic melanophore development.

Examination of *erbb3b* and *tuba8l3a* mutants provides additional support for the idea that extra-hypodermal, nerve-associated precursors are essential for metamorphic melanophore development. We found that presumptive precursors to glia and pigment cells were missing from *erbb3b* mutants and wild-type larvae in which ErbB activity had been inhibited during the embryonic critical period for adult melanophore development. An on-going requirement for *erbb3b* is suggested as well, by reduced complements of adult melanophores following acute inhibition of ErbB signaling in sensitized backgrounds during pigment pattern metamorphosis [43], and by reduced rates of EdU incorporation in *erbb3b* mutants during post-embryonic development (this study). Because ErbB signaling can repress melanocyte differentiation [62], [73], a further role in preventing the premature differentiation of nerve-associated precursors to hypodermal pigment cells seems likely. Notably, our finding that extra-hypodermal, kitla-responsive melanogenic cells were missing in ErbB-deficient backgrounds at post-embryonic stages contrasts with increased numbers of such cells at embryonic/early larval stages [74]. This difference likely reflects the pleiotropic nature of ErbB signals and a difference between the stages examined: blocking ErbB activity in the embryo presumably results in a de-repression of melanophore differentiation amongst transiently persisting precursor cells, whereas by post-embryonic stages, such precursors presumably have been lost.

In contrast to *erbb3b* mutants, *tuba8l3a* mutants exhibit a post-embryonic demyelination of peripheral nerves and a corresponding critical period for adult melanophore development [36], [44], [45]. In agreement with our model, *tuba8l3a* mutant larvae were deficient for mbp+ glia, nerve-associated foxd3+ cells and foxd3+; mitfa::GFP+ cells, and also had reduced rates of division amongst hypodermal mitfa::GFP+ cells. Because *tuba8l3a* acts autonomously to the metamorphic melanophore lineage [45], these findings suggest a defect in the maintenance or expansion of pluripotent precursors to mbp+ glia and mitfa:GFP+ pigment cell precursors. The post-embryonic onset of these phenotypes further suggests the existence of genetically distinct populations of embryonic and adult glia, analogous to embryonic and metamorphic melanophores.

### Pattern development, regulation, and the roles of temporally and genetically distinct metamorphic melanophore populations

Time-lapse imaging comparisons of wild-type and mutant backgrounds further defined roles for genes previously known to function in pigment pattern development: *kita*, *csf1r*, and *ednrb1* all promoted the survival of mitfa::GFP+ cells whereas *kita* also promoted the differentiation of these cells as melanophores. Yet, these analyses revealed compensatory responses of pigment cells and their precursors in mutant backgrounds as well. For example, residual mitfa::GFP+ cells in mutants with extra-hypodermal precursor deficiencies (*erbb3b*, *tuba8l3a*) or hypodermal defects in cell survival (*csf1r*, *ednrb1*) exhibited concomitantly greater rates of differentiation, and, in one instance (*erbb3b*), an increased rate of proliferation. Moreover, the reduced survival and differentiation of mitfa::GFP+ cells in *kita* mutants was coupled with a 70-fold increase in melanophore proliferation. These findings highlight the remarkably regulative nature of zebrafish pigment pattern development [36], [41], [42] and also the importance of direct imaging for understanding cellular behaviors that would not be predicted from terminal phenotypes alone.

A particularly dramatic example of pattern regulation occurs during regeneration. Zebrafish larval melanophores regenerate following laser ablation or the administration of melanocytotoxic drugs [74]–[76], adult fin melanophores regenerate along with other tissues after fin amputation [77]–[79], and hypodermal body melanophores regenerate after localized laser ablations [80], [81]. To test the capacity of latent precursors to supply new hypodermal melanophores in the adult, we used fish doubly mutant for *kita* and *csf1r^TS^* in which loss of residual melanophores at restrictive temperature likely reflects the withdrawal of trophic support provided by *csf1r*-dependent xanthophores [42], [60]. Our finding that progressively fewer hypodermal melanophores were recovered after repeated ablations implies a limited re-population potential for latent precursors that give rise to these *kita*-independent, *csf1r*-dependent late metamorphic melanophores. Whether the same is true of *kita*-dependent early metamorphic melanophores remains to be determined. Nevertheless, our finding that scale melanophores regenerated repeatedly-in contrast to late metamorphic, hypodermal melanophores-suggests a more highly regulative precursor pool associated with the adult scales, and highlights the possibility of spatially and temporally distinct pools of precursors having different morphogenetic and differentiative potentials. That scale melanophores developed *de novo* in these fish, despite their absence from *kita* single mutants, likely reflects a priority effect; e.g., initially abundant xanthophores may repress melanophore development in *kita* mutants [42], [82], [83] but simultaneous development of both melanophores and xanthophores during regeneration allows for a stable pattern comprising both cell types. The similar re-population potential of scale and fin melanophores, and the previous observation that *basonuclin-2* mutants, which are deficient for hypodermal melanophores, nevertheless retain both scale and fin melanophores [84], also suggest the possibility of more extensive similarities between scale- and fin-associated precursor pools.

Finally our study provides new insights into the temporally distinct populations of melanophores in zebrafish. We found severe deficiencies in the numbers of extra-hypodermally derived mitfa::GFP+ cells in *erbb3b* and *tuba8l3a* mutants, illustrating the critical role of such cells in supplying metamorphic melanophores overall. By contrast, we found no evidence for reduced numbers of extra-hypodermally derived mitfa::GFP+ cells in *kita*, *csf1r*, or *ednrb1* mutants, indicating that different genetic requirements of early and late metamorphic melanophores do not reflect differences in the establishment or maintenance of these cells. These findings suggest either of two interpretations. Early and late metamorphic melanophores could arise from a common precursor pool with differences in residual melanophore complements among mutants reflecting specific requirements for *kita*, *csf1r*, and *ednrb1* in downstream events of morphogenesis and differentiation. For example, our data indicate that mitfa::GFP+ cells require *kita* for their surival, and presumably, terminal differentiation: the development of late metamorphic melanophores in *kita* mutants could thus reflect the late appearance of factors able to substitute for *kita* activity in promoting melanophore differentiation. Conversely, we found that mitfa::GFP+ cells were only marginally dependent on *csf1r*: the failure of late metamorphic melanophores to develop in *csf1r* mutants could, in turn, reflect a late-arising, post-differentiation requirement for trophic support from *csf1r*-dependent xanthophores [38], [42], [60]. An alternative interpretation is that early and late metamorphic melanophores do arise from distinct, but still cryptic, precursor pools that simply were not revealed by our methods. In other systems, niches initially assumed to have just one type of stem or progenitor cell have sometimes been found to harbor distinct classes of cells with disparate proliferative or differentiative potentials [85]–[87]. Additional time-lapse imaging analyses at later stages and genetically based lineage analyses that are now being conducted should provide further insights into these possibilities.

### Post-embryonic stem cells in development, evolution, and neoplasia

The identification of extra-hypodermal nerve-associated precursors to adult melanophores in zebrafish (this study) and amniotes [62] indicates that a fuller understanding of adult pigment cell development and pattern formation requires a focus on post-embryonic precursors, as distinct from embryonic neural crest cells. The existence of genetically separable populations of embryonic pigment cells, derived directly from neural crest cells, and adult pigment cells, derived from post-embryonic precursors, further suggests that species-differences in adult pigment patterns may be explicable by evolutionary changes in the establishment, maintenance or recruitment of post-embryonic latent precursors, with few if any consequences for earlier pigment patterns [88]. Lastly, a peripheral nerve origin for adult pigment cells also raises the possibility that the frequent and generally fatal metastases of melanoma cells to the central nervous system [26]–[29],[89],[90] may reflect the continued or reiterated expression of genes that favor proliferation and migration in a nerve microenvironment.

## Materials and Methods

### Fish rearing, staging, and genetic stocks

Fish were reared at 28–29°C, 14L:10D. Post-embryonic stages are reported as standardized standard length (SSL) measurements, which indicate developmental progress of free-feeding larvae more reliably than days post-fertilization [33]. *Tg(mitfa::GFP)^w47^* and *Tg(−4.9sox10:egfp)^ba2^* fish were generously provided by D. Raible and R. Kelsh, respectively. *Tg(TrDct::mCherry)^wp.r.t3^* and *Tg(hsp70::kitla)^wp.r.t2^* fish were produced using tol2kit Gateway vectors and *Tol2-*mediated transgenesis [91], [92] and heat shocks with the latter strain were administered at 37°C for 1 hr three times daily for two days. Experiments with *erbb3b* mutants used either of two presumptive null alleles, *erbb3b^ut.r2e1^* or *erbb3b^wp.r2e2^* [93]. Experiments with *csf1r* mutants used either the presumptive null allele *csf1r^j4blue^* or the temperature-sensitive allele *csf1r^ut^* ^.*r1e174*^ [60]. In temperature shift experiments, fish were shifted repeatedly between restrictive temperature (33°C) and permissive temperature (24°C). All experiments with the *kita* mutant used the presumptive null allele *kita^b5^* [59]. *tuba8l3a^j115e1^* encodes a missense substitution with temperature-sensitive effects. Experiments with this allele were performed at standard rearing temperature, intermediate between restrictive (33°C) and permissive (24°C) temperatures [36], [44], [45], to allow analyses of a fuller complement of mitfa::GFP+ cells. Quantitative analyses here are thus likely to underestimate effects of the *tuba8l3a* mutation. Animal use conformed to University of Washington IACUC guidelines.

### Imaging and image analysis

Fish were viewed with Olympus SZX-12 or Zeiss Discovery epifluorescence stereomicroscopes or with a Zeiss Observer inverted compound epifluorescence microscope with Apotome. Images were collected in Axiovision software using Axiocam HR or MR3 cameras. For thick specimens, stacks of images were collected and processed using Zeiss Axiovision 6D Acquisition or Extended Focus modules and some fluorescence images were deconvolved using the Zeiss Axiovision Deconvolution module. Alternatively, specimens were viewed and images collected on Zeiss 510 META or Olympus FV1000 laser confocal microscopes.

### Histology

For immunohistochemistry, larvae were fixed in 4% paraformaldehyde containing 1% DMSO in PBS, rinsed, embedded in agarose, then sectioned by vibratome at 150–200 µm. Sections were washed in PBS/1% DMSO/0.3% Triton-X pH 7.4 (PDTX), blocked in PDTX containing 10–20% heat inactivated goat serum then incubated overnight at 4°C with primary antibody. We used polyclonal antisera raised in rabbit against zebrafish sox10 (1:500; provided by B. Appel [94]), zebrafish foxd3 (1:400; D. Raible [47]), zebrafish mbp (1:50; W. Talbot [95]), and GFP (1:200; A11122, Invitrogen) as well as monoclonal antibodies against GFP (1:200; 3E6 A11120, Invitrogen) and acetylated α-tubulin (1:200; 6-11B-1, T6793 Sigma). After washing, sections were incubated with secondary antibodies (AlexaFluor 405, 488, 568, 647; Invitrogen), washed and imaged.

In situ hybridization of post-embryonic zebrafish followed [44], [84]. For some analyses larvae were sectioned at 100–300 µm by vibratome. Detailed methods for in situ hybridization are available online at http://protist.biology.washington.edu/dparichy/.

### DiI injection

Cell Tracker CM-DiI (Invitrogen) was prepared as a stock solution in DMSO then diluted to 0.025–0.05% in 0.3 M sucrose just before use. Larvae were anesthetized briefly and injected with 1–2 nl of DiI using a borosilicate needle, imaged immediately to ascertain the specificity of staining in target tissues, then reared individually until analyzed.

### *Ex vivo* time-lapse imaging

Larvae were rinsed with 10% Hanks medium, anesthetized and then sacrificed by decapitation using a razor blade. After removing the anterior portion of the trunk and discarding the tails, larval trunks were placed on 0.4 µm transwell membranes (Millipore) in glass-bottom dishes containing L-15 medium, 3% fetal bovine serum, and penicillin/streptomycin. Trunks were equilibrated at 28.5°C for 3 h then imaged for 18–24 h (20 or 30 minute intervals between images) on a Zeiss Observer inverted epifluorescence microscope. Comparisons between isolated trunks imaged continuously for 24 h and repeatedly anesthetized intact larvae did not reveal gross differences in the survival of mitfa::GFP+ cells, though average maximal estimated velocities of mitfa::GFP+ cells were reduced by ∼22% in cultured trunks as compared to intact larvae (*P*<0.05; *N* = 67 cells examined). Imaging over longer durations resulted in increased rates of cell death throughout the explant and thus were not used for analyses.

### Pharmacological inhibition of ErbB activity

A stock solution of AG1478 [4-(3-chloroanilino)-6,7-dimethoxyquinazoline; Calbiochem] was diluted in DMSO. Embryos were treated with 3 µM AG1478 in 10% Hanks for through either 72 or 96 hours post-fertilization. To facilitate penetration, 0.5% DMSO was added to all media and embryos were dechorionated prior to treatment. Fish were reared in glass Petri dishes and solutions were changed daily.

### EdU labeling

Larvae were incubated with 0.005% 5-ethynyl-2′-deoxyuridine (EdU; Intvitrogen) in 10% Hank's medium containing 1% DMSO for 36 h. Larvae were then sacrificed, fixed with 4% PFA/1% DMSO, and vibrotome sectioned (150–200 µm) for immunohistochemistry followed by histochemical detection of EdU according to manufacturer's recommendations.

### Statistical analyses

Quantitative data were analyzed with JMP 8.0.2 (SAS Institute, Cary NC). Frequency data were examined using multiple logistic regression or contingency table analyses, and tested for effects of genotype, stage, and genotype x stage interactions. Significance of effects were assessed by likelihood ratio tests and non-significant factors were removed from the final models. Analyses of variance were used for continuous variables including counts. Residuals were examined for normality and homogeneity of variances, conditions that were achieved for some variables after transformation by square root or natural logarithm. Further details of statistical analyses are available upon request.

## Supporting Information

Figure S1 mitfa::GFP+ cells amongst glia of the lateral line nerve. Sagittal view of a wild-type larva showing mitfa::GFP+ cells (mitfa, green; arrow) aligned with mbp+ glia (red) of the main trunk lateral line nerve, near the horizontal myoseptum.
(TIF)

Figure S2 Ectopic kitla-responsive melanogenic cells were nerve-associated. Shown is an ectopic sox10+ (red) melanophore (arrow) within the myotome adjacent to a nerve fiber stained for acetylated tubulin (green).
(TIF)

Figure S3 Defects in wild-type larvae treated with ErbB inhibitor AG1478 during the *erbb3b* embryonic critical period. (A,B) Post-embryonic mbp+ glia (red; arrowheads) were reduced though not eliminated in AG1478-treated larvae. Inset, mitfa::GFP+ cell (green) aligned on mbp+ glia of a peripheral nerve at the level of the horizontal myoseptum. sc, spinal cord; m, myotome; e, epidermis; ll, lateral line nerve. (C,D) foxd3+ cells (red; arrowheads) within the myotome were missing from AG1478-treated larvae.
(TIF)

Figure S4 Deficiencies in mitfa::GFP+ cells, foxd3+ cells, and EdU incorporation in *erbb3b* mutants. (A,B) Views and annotations correspond to those for wild-type larvae in main text Figure 5. Arrow, a rare EdU+; mitfa::GFP+ cell at the base of the ventral fin.
(TIF)

Figure S5 *erbb3b*-dependence of *kita*-independent hypodermal melanophores. (A) *kita^b5^* presumptive null allele [59] with stripes of *kita*-independent hypodermal melanophores. (B) Fish doubly mutant for *kita^b5^* and the presumptive null allele *erbb3b^ut.r2e1^* showing loss of many *kita*-independent melanophores as well as gaps in the “interstripes” (e.g., arrowhead) reflecting an iridophore deficiency. Patches of residual melanophores may be of clonal origin.
(TIF)

Figure S6 mitfa::GFP+ cells in adult fish. Shown are cross-sections through ∼20 SSL (∼80 days post-fertilization) adult wild-type fish. (A) A persisting nerve-associated mitfa::GFP+ cell (green; arrow). sc, spinal cord; m, myotome. (B) mitfa::GFP+ cells (green; arrowhead) within the lateral line nerve. e, epidermis (C) mitfa::GFP+ cells (green; arrow) and foxd3+ cells (red; arrowhead) in the ventral myotomes and base of the anal fin (f). (D) mitfa::GFP+ cell (green; arrow) and doubly labeled mitfa::GFP+; foxd3+ cell (arrowhead) associated with an adult scale (s).
(TIF)

Video S1 mitfa::GFP+ cells in a living wild-type larva. Images were collected in sagittal planes from the skin to the midline of the trunk. Image rotation reveals extra-hypodermal cells within the myotomes, that typically expressed mitfa::GFP at levels lower than in hypodermal cells.
(MOV)

Video S2 mitfa::GFP+ and dct::mCherry+ melanophores in a living wild-type larva. In contrast to mitfa::GFP+ cells (green), cells expressing the later melanophore lineage marker *dct* were detected only in the hypodermis. Shown here are cells expressing mCherry driven by the *Takifugu rubripes dct* promoter.
(MOV)

Video S3 mitfa::GFP+ cells are missing from a live *erbb3b* mutant larva. Larva imaged as in Video S1 but over-exposed to provide spatial context. Fluorescent cells forming a stripe in the hypodermis are iridophores, which reflect under epifluorescence in multiple channels (these cells are present in the larva shown in Video S1, but their reflectance falls beneath the threshold for detection at the exposure shown).
(MOV)

Video S4 mitfa::GFP+ cells differentiate as melanophores in the hypodermis. mitfa::GFP+ cells acquiring melanin (arrows) over ∼18 h.
(MOV)

Video S5 :GFP+ cells. Low magnification view showing highly migratory mitfa::GFP+ cells over ∼18 h. This movie loops three times. mitfa::GFP+ cells are highly migratory and extend long probing processes. These cells can be seen migrating widely over the flank and also migrating from the body into the anal fin at the lower edge of the frame. In contrast to mitfa::GFP+ cells, xanthophores are larger, less motile and autofluoresce weakly in the GFP channel, particularly from accumulations of pteridine-containing organelles around the nucleus.
(MOV)

Video S6 Migration of mitfa::GFP+ cells between body and fin. Detail at the margin between body and anal fin, showing movements of mitfa::GFP+ cells between these regions.
(MOV)

Video S7 mitfa::GFP+ cells emerging from within the myotome. Several mitfa::GFP+ cells (arrows) enter the hypodermis from within the myotome; also see Video S8.
(MOV)

Video S8 mitfa::GFP+ cells emerging from within the myotome. High magnification view showing a single mitfa::GFP+ cell (arrow) emerging from within the myotome. In this movie, only selected planes of focus from a complete z-series are shown, illustrating the progressive movement of this cell from the interior myotome to the surface. As the cell approaches the more superficial planes, additional mitfa::GFP+ cells already in the hypodermis come into focus.
(MOV)

Video S9 mitfa::GFP+ cell emerging from the vicinity of the horizontal myoseptum. The movie loops three times.
(MOV)

Video S10 mitfa::GFP+ cells entering the hypodermis after migrating over the dorsal myotome. One cell already at the dorsal myotome and another extending a process over the flank at the beginning of the movie (red arrows). A third cell extends a long filopodium (yellow arrow) over the flank before entering into the hypodermis.
(MOV)

Video S11 mitfa::GFP+ cells migrating within the caudal nerve plexus. Shown are mitfa::GFP+ cells along a nerve plexus near the base of the caudal fin in the medial region of a larva. This particular individual is a *kita* mutant. Similar behaviors were observed in wild-type larvae.
(MOV)

Video S12 mitfa::GFP+ cells migrating along and dispersing from peripheral nerves. Shown is an enhanced detail of Video S11. Arrowhead indicates a cell traversing along a nerve fiber then migrating away.
(MOV)

Video S13 Morphogenesis of residual mitfa::GFP+ cells in *erbb3b* mutants. Residual mitfa::GFP+ cells were often patchily distributed and typically expressed GFP at lower levels than in wild-type. Red arrowhead, mitfa::GFP+ cell dividing. Arrow, mitfa::GFP+ cell emerging from the vicinity of the horizontal myoseptum. Yellow arrowhead, death of mitfa::GFP+ cell.
(MOV)

Video S14 Proliferation of mitfa::GFP+ cells. A mitfa::GFP+ cell (arrow) divides within the hypodermis over ∼14 h in a wild-type larva.
(MOV)

Video S15 Death of mitfa::GFP+ cells. Fragmentation and loss of three mitfa::GFP+ cells (arrows) in a *kita* mutant larva over ∼16 h (see text for details).
(MOV)

Video S16 Proliferation of differentiated melanophores. Shown is the posterior tail of a kita mutant larva, illustrating successive melanophore divisions. mitfa::GFP+ cells also can be observed migrating on the body and between the body and caudal fin.
(MOV)
