# Supplementary figures and images for: Post-Embryonic Nerve-Associated Precursors to Adult Pigment Cells: Genetic Requirements and Dynamics of Morphogenesis and Differentiation
Source: PLoS Genet. 2011 May 19;7(5):e1002044. doi: 10.1371/journal.pgen.1002044 (PMC3098192; doi:10.1371/journal.pgen.1002044)

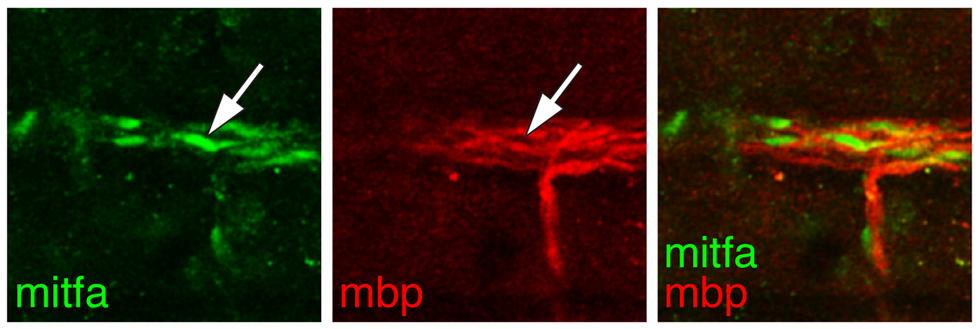

Supplement: Figure S1 — mitfa::GFP+ cells amongst glia of the lateral line nerve. Sagittal view of a wild-type larva showing mitfa::GFP+ cells (mitfa, green; arrow) aligned with mbp+ glia (red) of the main trunk lateral line nerve, near the horizontal myoseptum. (TIF) [file pgen.1002044.s001.tif]

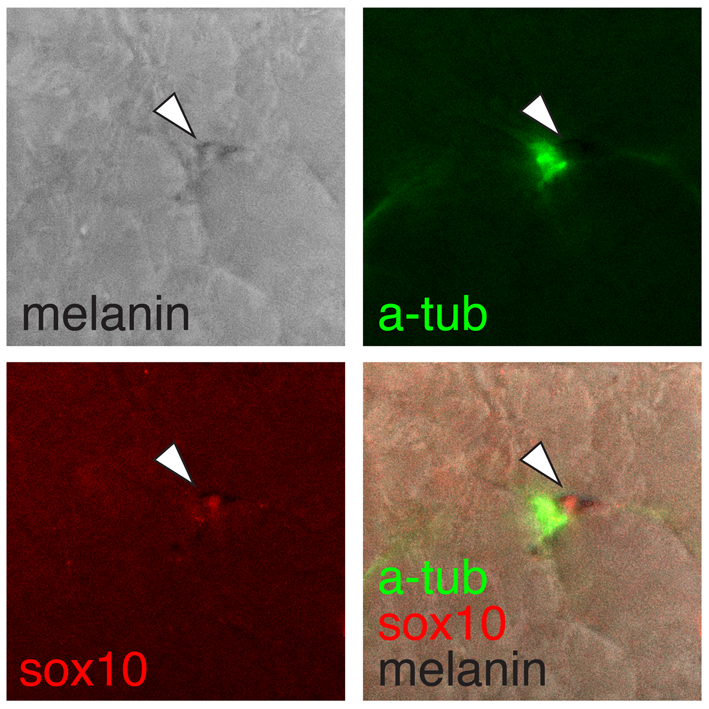

Supplement: Figure S2 — Ectopic kitla-responsive melanogenic cells were nerve-associated. Shown is an ectopic sox10+ (red) melanophore (arrow) within the myotome adjacent to a nerve fiber stained for acetylated tubulin (green). (TIF) [file pgen.1002044.s002.tif]

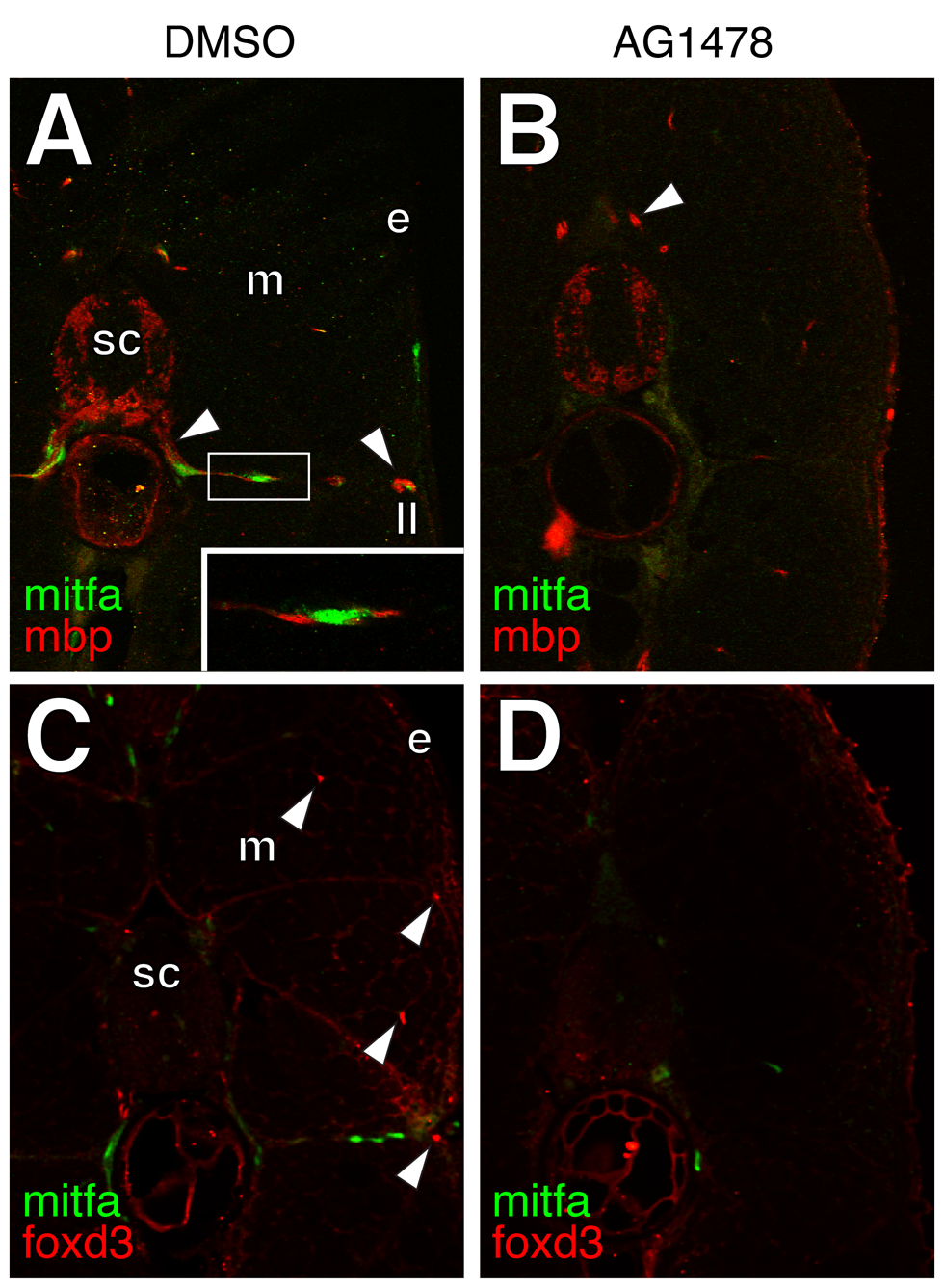

Supplement: Figure S3 — Defects in wild-type larvae treated with ErbB inhibitor AG1478 during the erbb3b embryonic critical period. (A,B) Post-embryonic mbp+ glia (red; arrowheads) were reduced though not eliminated in AG1478-treated larvae. Inset, mitfa::GFP+ cell (green) aligned on mbp+ glia of a peripheral nerve at the level of the horizontal myoseptum. sc, spinal cord; m, myotome; e, epidermis; ll, lateral line nerve. (C,D) foxd3+ cells (red; arrowheads) within the myotome were missing from AG1478-treated larvae. (TIF) [file pgen.1002044.s003.tif]

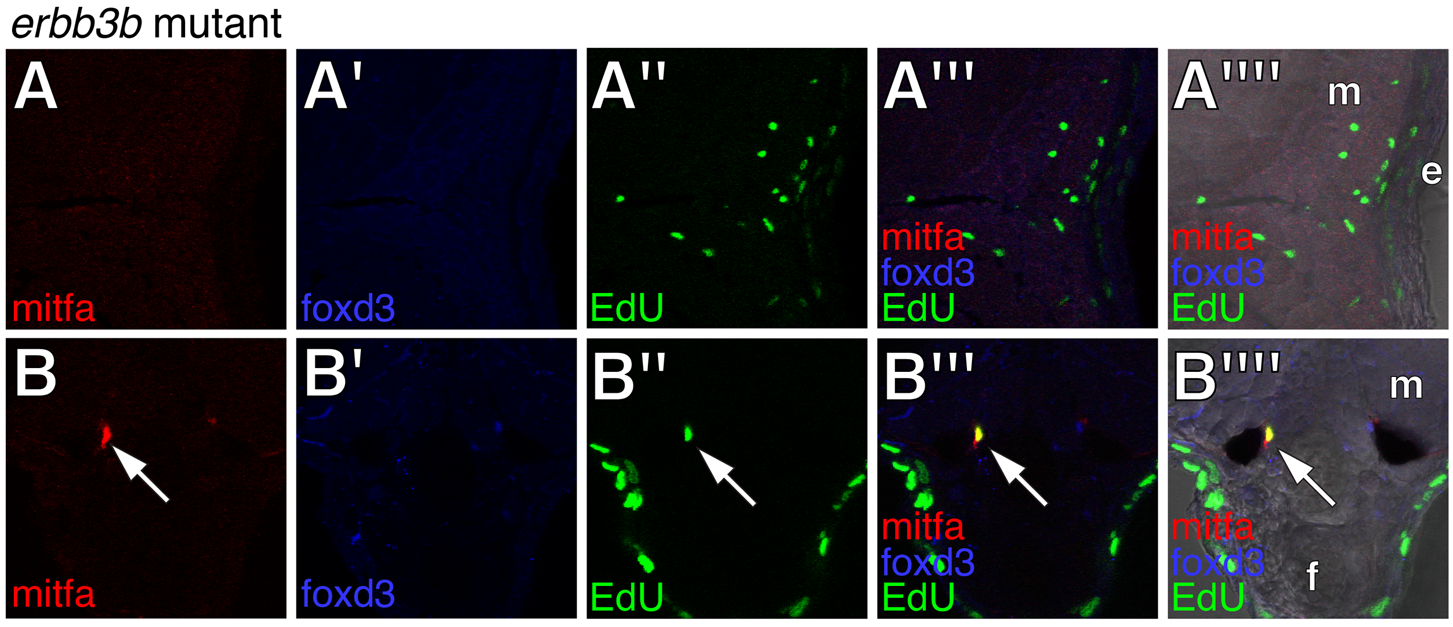

Supplement: Figure S4 — Deficiencies in mitfa::GFP+ cells, foxd3+ cells, and EdU incorporation in erbb3b mutants. (A,B) Views and annotations correspond to those for wild-type larvae in main text Figure 5. Arrow, a rare EdU+; mitfa::GFP+ cell at the base of the ventral fin. (TIF) [file pgen.1002044.s004.tif]

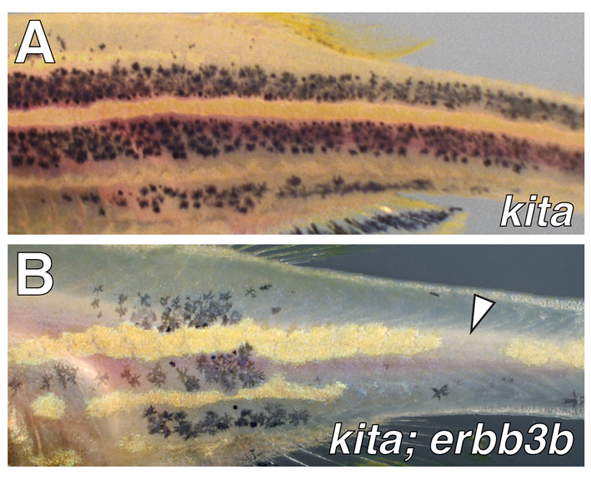

Supplement: Figure S5 — erbb3b-dependence of kita-independent hypodermal melanophores. (A) kitab5 presumptive null allele [59] with stripes of kita-independent hypodermal melanophores. (B) Fish doubly mutant for kitab5 and the presumptive null allele erbb3but.r2e1 showing loss of many kita-independent melanophores as well as gaps in the “interstripes” (e.g., arrowhead) reflecting an iridophore deficiency. Patches of residual melanophores may be of clonal origin. (TIF) [file pgen.1002044.s005.tif]

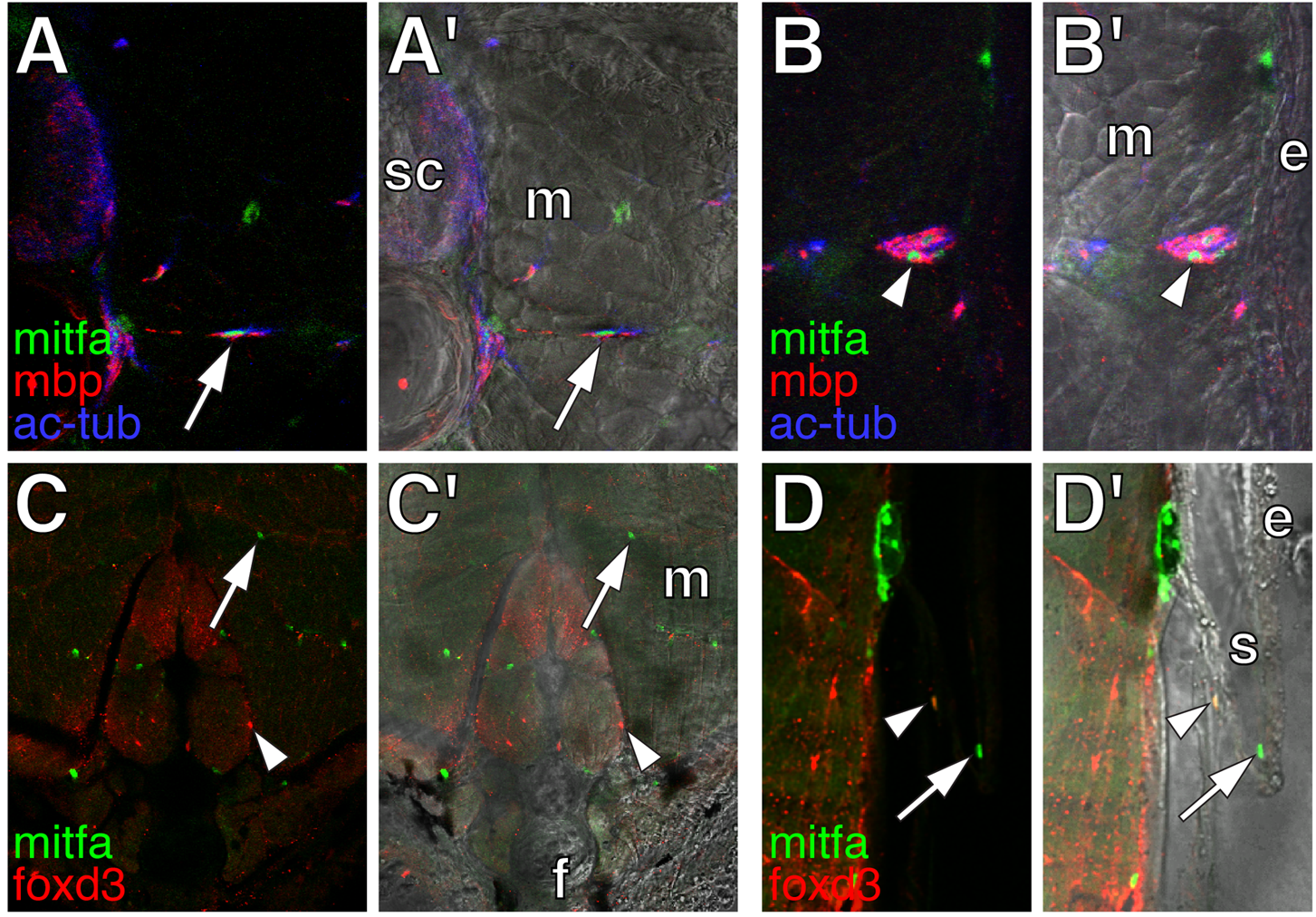

Supplement: Figure S6 — mitfa::GFP+ cells in adult fish. Shown are cross-sections through ∼20 SSL (∼80 days post-fertilization) adult wild-type fish. (A) A persisting nerve-associated mitfa::GFP+ cell (green; arrow). sc, spinal cord; m, myotome. (B) mitfa::GFP+ cells (green; arrowhead) within the lateral line nerve. e, epidermis (C) mitfa::GFP+ cells (green; arrow) and foxd3+ cells (red; arrowhead) in the ventral myotomes and base of the anal fin (f). (D) mitfa::GFP+ cell (green; arrow) and doubly labeled mitfa::GFP+; foxd3+ cell (arrowhead) associated with an adult scale (s). (TIF) [file pgen.1002044.s006.tif]
